# Synergistic Effects of MHD Dynamics and Oxygen Vacancies on Electrode Polarization in Photoelectrocatalysis CO_2_ Reduction Systems

**DOI:** 10.1002/EXP.20240243

**Published:** 2025-12-08

**Authors:** Lei Zhao, Feng Xiao, Xianghui Zeng, Zhaohui Huang, Wei Fang, Xing Du, Xuan He, Weixin Li, Daheng Wang, Hui Chen

**Affiliations:** ^1^ The State Key Laboratory of Refractories and Metallurgy Wuhan University of Science & Technology Wuhan P. R. China; ^2^ College of Materials Science and Engineering Hunan University Changsha P. R. China

**Keywords:** CuFeO_2_, CO_2_RR, magnetohydrodynamic (MHD), oxygen vacancy, photoelectrocatalysis

## Abstract

Photoelectrocatalysis reduction of CO_2_ into products such as CO and CH_4_ is an effective strategy for improving carbon utilization and advancing the development of renewable energy. Improving the catalytic efficiency by regulating the polarization behavior of the electrode has been proven to be an effective method. In this study, a method for preparing a Ti:Fe_2_O_3_/CuFeO_2_‐v photoanode with oxygen vacancies and heterojunctions for PEC CO_2_ reduction is reported. Oxygen vacancies not only enhance the carrier transport ability of the electrode and improve the resistance polarization, but also regulate the material's magnetic properties. Based on this, we utilize the magnetic fluid dynamics (MHD) effect to reduce the thickness of the diffusion layer on the electrode surface, thereby improving mass transfer and solving the concentration polarization problem. This adjustment increased the current density to 1.49 mA cm^−2^ and increased the CO yield to 154.27 mL h^−1^. This method innovatively applies the MHD insights of the electrochemical oxygen evolution reaction (OER) to photoelectrochemical CO_2_ reduction, aiming to optimize the electrode reaction kinetics for efficient CO_2_ conversion, marking a significant progress in the field of photocatalytic CO_2_ reduction.

## Graphical abstract

This study fabricated oxygen‐vacancy‐doped CuFeO_2_‐based photocathodes, achieving efficient CO_2_ conversion through the synergistic effects of light, electricity, and magnetic fields.

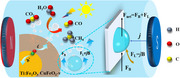

## 1 Introduction

The growing concerns about climate change and the urgent need to reduce carbon emissions have spurred research on strategies for reducing carbon dioxide (CO_2_) [1, 2]. Among these, photoelectrocatalytic reduction of CO_2_ to valuable hydrocarbons and oxygenates shows promise for both sequestering atmospheric CO_2_ and producing sustainable energy [3, 4]. However, these processes often suffer from slow reaction kinetics and low selectivity towards desired products [5, 6, 7].

In the pursuit of efficient photoelectrocatalysis, the selection of catalysts that meet thermodynamic requirements has been fundamental [8, 9]. However, it is increasingly recognized that the kinetics of these catalysts play a more influential role in determining overall performance [10, 11]. In particular, regulating kinetic behavior at the electrode surface is critical for enhancing catalytic efficiency [12]. This kinetic behavior, often assessed through polarization curves, is crucial for understanding and improving the photoelectrocatalytic system. Generally, electrode polarization behavior can be categorized into three types: resistance (ohmic), electrochemical polarization, and concentration polarization [13, 14]. Resistance polarization directly relates to the resistance of electrochemical reaction components and electrolyte. Electrochemical polarization behavior is commonly evaluated using Tafel curves. As shown in the following formula:

$$\eta =a+\text{blg}\left|j\right|$$

While *η* indicates overpotential, *j* is current density, a is transfer coefficient, b is Tafel constant. This formula is the most widely used formula to calculate the dynamic behavior of electrodes. The generation of electrochemical polarization is related to the hysteresis of electrode surface reaction, so optimizing the rate of electrode reaction can effectively improve the effect of electrochemical polarization.

However, the application of the Tafel formulation is limited, and the formula cannot be used if the electrode has concentration polarization. In this case, we can continue to study the dynamics of the electrode surface by using the Butler–Volmer formula:

$$\varepsilon =\frac{RT}{\beta F}\;\ln\frac{I_{d}}{I_{d}-I}$$

While the limiting current density (*I*_d_) is related to the diffusion layer (*δ*) according to Fick's first law:

$$\;I_{d}=nFD_{i}\frac{c_{i}^{B}}{\delta }\left(\;c_{i}^{S}=0\right)$$

*D*_i_ is the ion diffusion coefficient of component i, $c_{i}^{B}$ is the concentration of i in bulk solution, $c_{i}^{S}$ is the concentration of i on the electrode surface. It can be inferred from the formula that in order to improve the concentration polarization, it is necessary to enhance the mass transfer rate of the electrode surface or reduce the thickness of the diffusion layer (*δ*).

In terms of resistance and electrochemical polarization regulation, various strategies have been effective: (1) construct heterojunction, (2) creation of doping vacancies, and (3) development of unique morphologies and structures [15, 16, 17, 18, 19, 20, 21, 22, 23]. Scholars have conducted a lot of research in these fields and achieved many remarkable results. In 2022, Kan et al. developed a Si/ZnO/Cu_2_O p‐n‐p heterojunction that selectively reduces CO_2_ to ethanol with a Faradaic efficiency exceeding 60% at 0 V versus RHE, utilizing n‐type ZnO nanosheets to trap photogenerated electrons in a potential well formed between defect‐rich p‐type Cu_2_O and porous p‐type Si [24]. However, the adjustment of concentration polarization within the realm of photocatalytic CO_2_ reduction remains less explored.

**SCHEME 1:**
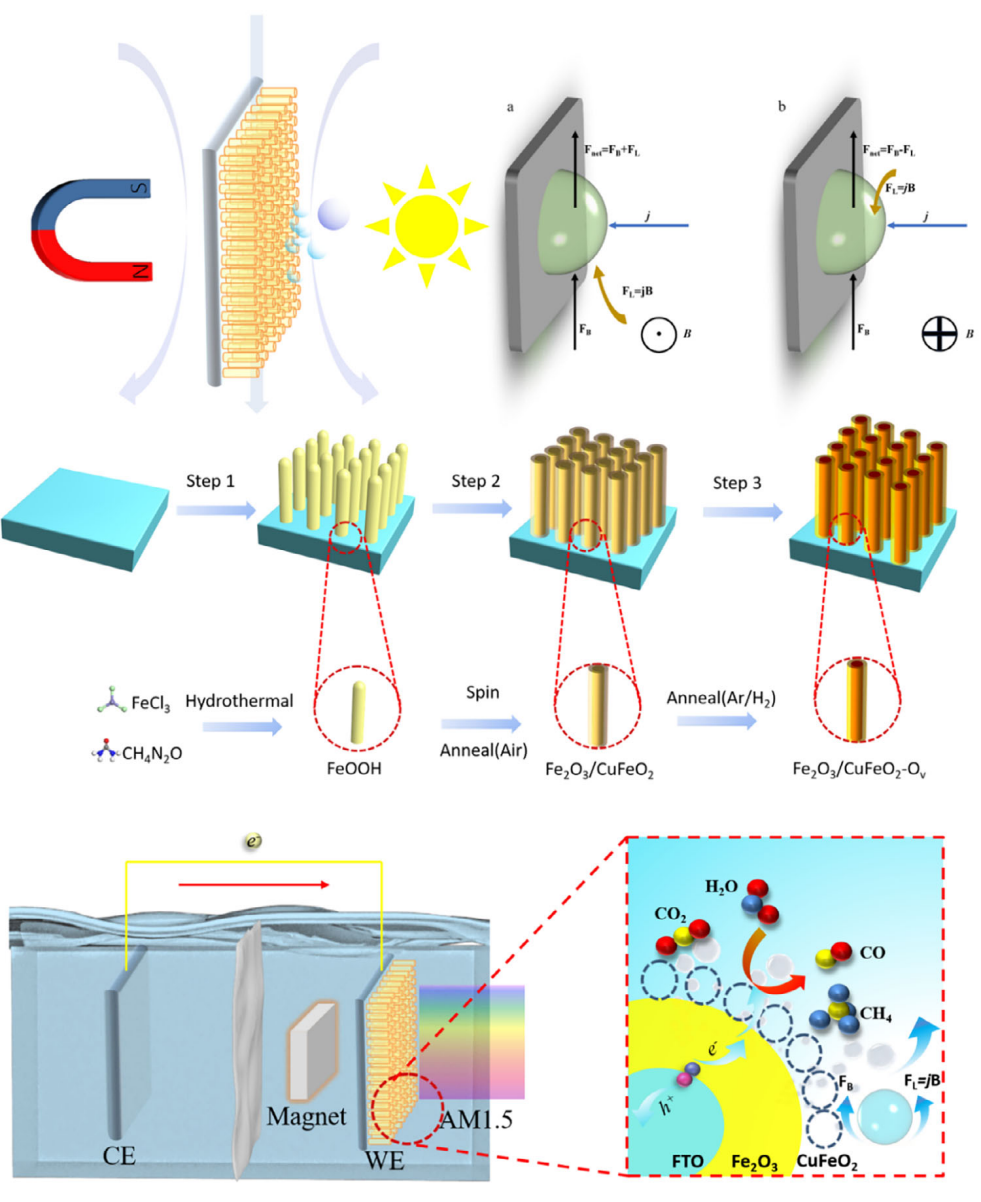
Synthesis route and reaction diagram.

Recent studies electrochemical oxygen evolution reaction/hydrogen evolution reaction (OER/HER) reveal that the magnetohydrodynamic (MHD) effect can reduce diffusion layer thickness, thereby enhancing mass transfer and ameliorating concentration polarization [25, 26]. The MHD effect predominantly influences gas‐evolving electrodes, where bubble release size and residence time depend on the net magnetic force (*F*_net_), as demonstrated in Scheme 1. If the direction of *F*_L_ and buoyancy force (*F*_B_) are the same (*F*_net_ = *F*_L_ + *F*_B_), the upward pumping effect of MHD convection will reduce the average size of bubble detachment, shorten the remaining time on the electrode, and increase the rising speed, while the opposite phenomenon will occur when *F*_L_ goes downward (*F*_net_ = *F*_L_ − *F*_B_). Apparently, the macroscopic MHD effect will bring about enhanced response of the gas evolving electrodes. Besides, the forced flow produced by MHD was inclined to impair the thickness of the diffusion layer (*δ*) and enhance mass transfer, thus increasing *I*_d_ and weakening the concentration polarization. For instance, Zheng et al. (2021) demonstrated that using NiCoFe‐MOF‐74 as an electrocatalyst in an alternating current (AC) magnetic field enhanced alkaline OER activity at low current densities [27]. However, its application in photoelectrochemical CO_2_ reduction has not been investigated. This study focuses on the applicability of MHD effects in photoelectrochemical CO_2_ reduction reactions (CO_2_RR).

Building on this foundation, our study utilizes a strategy involving the construction of heterojunctions and oxygen vacancies to synthesize Fe_2_O_3_/CuFeO_2_‐v thin‐film photocathode materials replete with oxygen vacancies. This approach aims to meticulously optimize electrode reaction kinetics, addressing both resistance and concentration polarization, thereby realizing an efficient photocatalysis CO_2_ reduction process.

## 2 Experimental Section

### 2.1 Material Information

The reagents used in this experiment include ferric chloride hexahydrate, urea, aluminum carbotitanate, sodium hydroxide, citric acid, ferric nitrate 9‐hydrate and copper nitrate trihydrate, all purchased from Chinese medicine reagents, the purity of the reagents is AR, and they have not been treated except aluminum carbotitanate before use.

### 2.2 Characterization

The powder X‐ray diffraction (XRD) patterns were operated on a Philips X'Pert Pro diffractometer by Ni‐filtered Cu Kα radiation (*λ* = 0.154056 nm) with Bragg diffraction angle between 5°–90°. The Fourier transform Raman spectra were collected using a Thermo Fisher DXR2xi 5225 microscope with a DXR 532 nm laser. X‐ray photoelectron spectroscopy (XPS) measurements were studied by a VG Multilab 2000 instrument (Thermo Electron Corporation) with Al Kα radiation. The field emission scanning electron microscope (SEM) images were observed on a FEI Company Novo 400 device with a voltage of 15 kV. The energy dispersive X‐ray (EDS) element mappings were scanned on a Thermo Electron Corporation Noran 623M‐3SUT spectroscope. The transmission electron microscopy (TEM) images were characterized by a JEOL JEM‐1400Plus at an accelerating voltage of 100 kV. UV–visible diffuse reflectance (UV–vis) spectroscopy was recorded using a Shimadzu UV‐2600 spectrophotometer with a transparent FTO glass subtracting the background. The steady‐state photoluminescence (PL) emission spectra were tested on Shimadzu RF‐6000, and transient fluorescence spectrometer was conducted by FLS‐980 Spectrometer (Edinburgh Instruments Ltd.) at room temperature. The variable temperature fluorescence test was performed using the FLS‐980 instrument from Edinburgh, UK, at room temperature or 150–300 K. The fluorescence decay plots were measured employing a pulsed excitation laser of 280 nm and detected on a high‐speed red. The vibrating sample magnetometer (VSM) test was performed at room temperature using the 7404 instrument of LakeShore, USA. Electron paramagnetic resonance (EPR) tests are performed at room temperature using a German Bruker EMX PLUS instrument. Atomic force microscopy (AFM) test was performed at room temperature using Bruker Dimension Icon.

### 2.3 Photo‐Electrochemical Measurements

Photo‐electrochemical (PEC) measurements were carried on CHI660E electrochemical workstation equipped with a standard three‐electrode configuration, adopting obtained photoanode as a work electrode, the Pt foil as a counter electrode and the Ag/AgCl electrode in 1 m NaOH as a reference electrode. CFO, TF, TF/CFO, and TF/CFO‐v films were carefully scraped into photoanodes with a geometric area of 0.5 cm × 0.5 cm by a squeegee blade. A fresh 1 m NaOH solution purified by nitrogen for 30 min was applied as the electrolyte medium, A 500 W Xenon lamp equipped with an AM 1.5 G filter (Zolix) was utilized as the light source with an irradiance intensity of 100 mW cm^−2^ calibrated by an NREL‐standard Si solar cell.

The incident photon‐to‐current conversion efficiency (IPCE) was tested by the Zennium C‐IMPS system (Zahner, TLS‐03) with wavelength from 365 to 700 nm at a bias of −0.7 V versus Ag/AgCl. Mott–Schottky (M–S) analysis was recorded on a Zennium photo‐electrochemical instrument (Zahner IM6 and PP211, Germany), in which the AC amplitude is −0.7 V and the frequency is 10 KHz. IMPS and IMVS was also tested by the Zahner system, with frequency from 10 to 50 Hz at the illumination intensity from 100 to 500 W, respectively.

The photocatalytic performance of the samples was tested using a three‐electrode system. The platinum electrode was used as the counter electrode, the saturated Ag/AgCl electrode as the reference electrode and the prepared samples as the working electrode. The reaction was carried out at room temperature and pressure. The CFO, TF, TF/CFO, and TF/CFO‐v were prepared into a working electrode with a geometrical area of 0.25 cm^2^ by using a scraper. The reaction vessel is a closed photocatalytic reaction cell. A freshly prepared 1 m aqueous solution of NaOH (pH = 14) was used as the electrolyte and purified with nitrogen for 30 min before the test to remove dissolved oxygen. Then, the reaction container is closed, irradiated under 500 W xenon lamp, and the bias voltage of −0.7 V is applied to start the reaction. The CO_2_RR produced and its output were analyzed and measured by the GC7900 gas chromatograph (CO_2_ carrier gas, FID detector) of Shimadzu Company in Japan. During the test, 1 mL gas from the top of the reactor was extracted to the gas chromatograph. The oxygen production efficiency of the catalyst can be obtained by converting the peak area obtained with the peak area obtained with standard oxygen. The single high frequency impedance test was performed using a CHI760E electrochemical workstation and a high‐speed camera to capture and test the cross section of the sample under the condition that the front section of the sample was illuminated.

Except as otherwise mentioned, the working electrodes were soaked for 30 min to fully contact with the electrolyte and all the above PEC tests were completed under front‐side irradiation. An interval period was kept for 20 min to return to a steady state after each photo‐electrochemical measurement.

## 3 Result and Discussion

### 3.1 Structural and Morphological Characterizations

**FIGURE 1:**
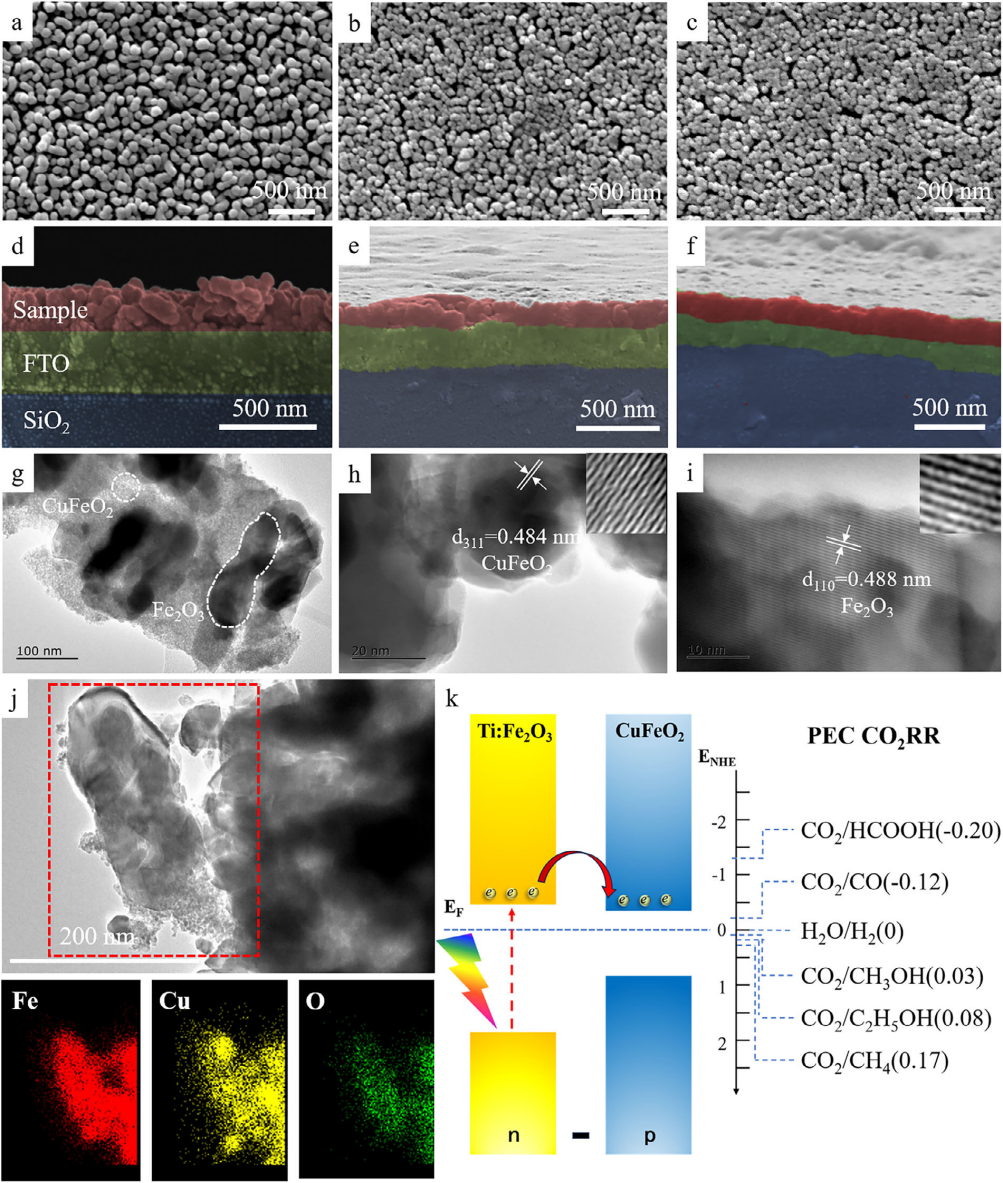
(a–c) SEM top view and, (d–f) SEM cross‐section photo of TF, TF/CFO, and TF/CFO‐v (Blue is the SiO_2_ layer, green is the FTO layer, and red is the sample layer); TEM photograph of (g–i) TF/CFO‐v; (j) EDS spectra of TF/CFO‐v, (k) band structure diagram of TF and CFO.

The morphological features of CFO, TF, TF/CFO, and TF/CFO‐v samples were examined in detail using a scanning electron microscope (SEM), as shown in Figure 1a–f. Unlike the observed agglomeration morphology of CFO samples (Figure S1), the TF samples exhibit a unique rod‐like arrangement, with nanorod diameters ranging from 30 to 50 nm, as shown in Figure S2 by EDS energy‐dispersive X‐ray spectroscopy. The film thickness of the sample can be judged to be approximately 230 nm (red area marked). It is worth noting that the TF/CFO and TF‐CFO‐v samples exhibit a composite structure, with CuFeO_2_ particles of approximately 20–30 nm stacked on the surface, and there was no significant difference between TF/CFO and TF‐CF‐v morphology. This unique arrangement suggests that CFO nanoparticles preferentially nucleate at the tip of the TF nanorods, leading to the observed morphology.

Transmission electron microscopy (TEM) analysis, as shown in Figure 1g–i, provided further insight into the nanostructure. Two different morphologies of materials, namely nanorods with a length of about 200 nm and a diameter of about 50 nm, and nanoparticles, were observed in the TF/CFO‐v sample. According to the images in the high resolution transmission electron microscopy (HRTEM), the lattice spacing of the nanorods was *d* = 0.488 nm, corresponding to the (110) crystal plane of Fe_2_O_3_, while the lattice spacing of the nanoparticles was *d* = 0.492 nm, related to the (220) crystal plane of CuFeO_2_ (consistent with TF and CFO in Figure S3). Further, the polycrystalline diffraction patterns of CFO, TF, and TF/CFO samples were shown in Figure S3c,f,i, where the polycrystalline circular pattern of CFO particles and the single crystal spot of TF were mixed together, and the EDS energy spectrum in Figure 1j also further proved the heterojunction relationship between TF and CFO, indicating the successful synthesis of the TF/CFO composite material. In order to explore the electron transfer path between TF and CFO, the energy band structure of the sample was characterized (Figure S4). According to the slope of the MS curve in Figure S5, it can be determined that TF and CFO are n‐type and p‐type semiconductors respectively. In theory, a p‐n junction can be formed as shown in Figure 1k. Based on this, electrons excited by sunlight will be transferred from TF to the lower level CFO and then migrate to the surface for CO_2_ reduction reaction.

### 3.2 Photocatalytic Performance for CO_2_ Reduction

**FIGURE 2:**
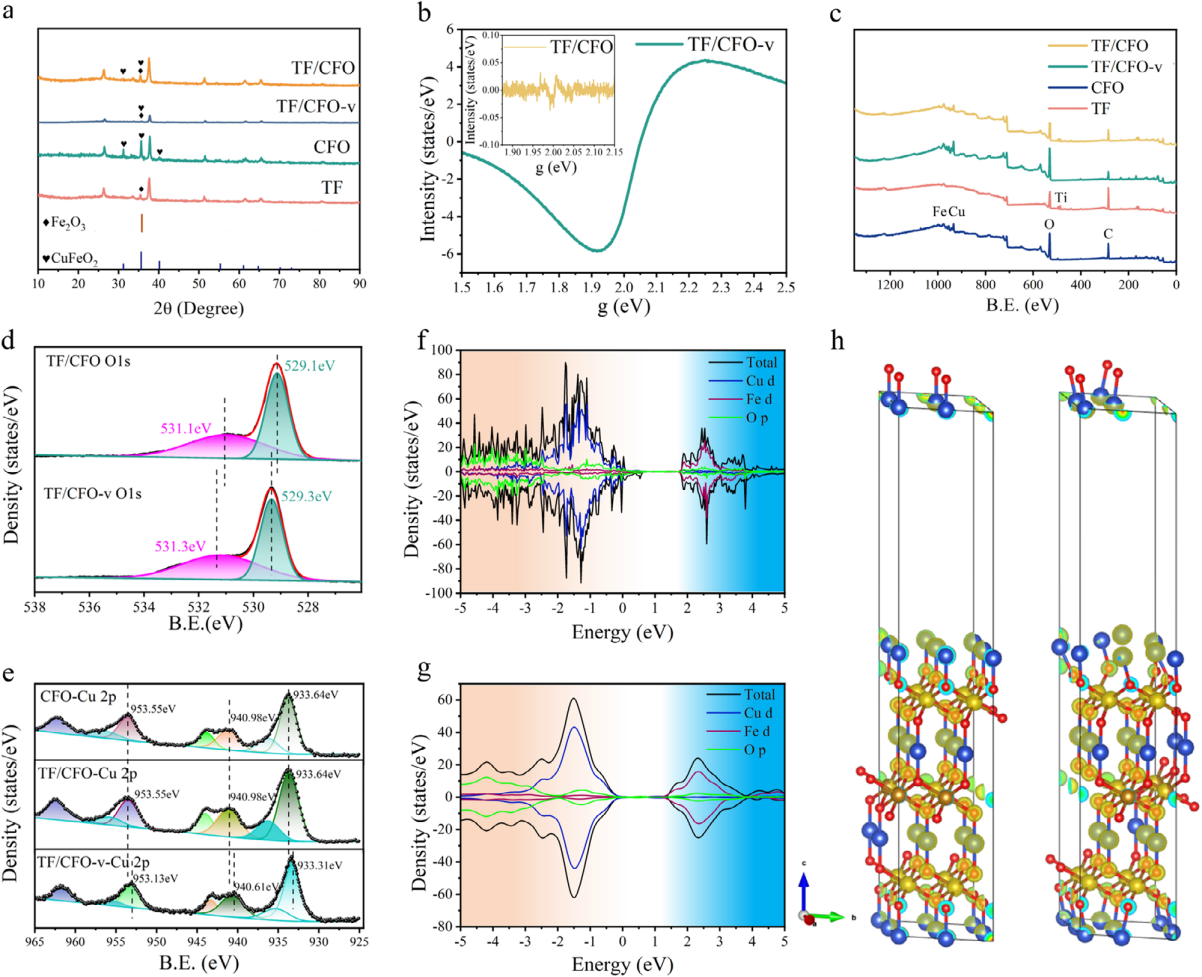
(a) XRD patterns, (b) EPR patterns and XPS spectrum of samples: (c) Total spectrum, (d) O 1s spectrum, (e) Cu 2p spectrum. Calculated DOS of (f) CuFeO_2_ slab with oxygen vacancy and (g) CuFeO_2_ slab. The partial charge density around the valence band maximum (marked with the orange region) of (h) CuFeO_2_ slab and CuFeO_2_ slab with oxygen vacancy.

Figure 2a presents the X‐ray Diffraction (XRD) pattern of CFO, TF, TF/CFO, and TF/CFO‐v samples. It confirms the hematite phase of the TF sample, characterized by distinct diffraction peaks at 2*θ* = 35.7° and 33.1°, corresponding to the (110) and (104) planes, respectively. Moreover, the original crystal structure of hematite is retained post introduction of Ti ions. Similarly, the CFO sample exhibits characteristic diffraction peaks at 2*θ* = 35.5° and 30.1°, indexed to the (311) and (220) planes of CuFeO_2_. For TF/CFO and TF/CFO‐v, the characteristic peaks of both CFO and TF phases are simultaneously present, confirming the coexistence of these phases in the composites. It is worth noting that, compared to TF/CFO, the peak shape of TF/CFO‐v is sharper and the peak position has shifted slightly towards higher values. This may be due to improved crystallinity caused by secondary annealing, as well as a decrease in interplanar spacing resulting from the introduction of oxygen vacancies (Figure S6). Electron paramagnetic resonance (EPR) mapping, as shown in Figure 2b, further substantiates the successful introduction of oxygen vacancies. The significant increase in the peak value at *g* = 2.0 for the TF/CFO‐v sample post Ar/H_2_ gas treatment is a clear indicator of oxygen vacancy formation. X‐ray photoelectron spectroscopy (XPS) analyses were conducted to characterize the elemental chemical states within the samples. As depicted in Figure 2c, the XPS full‐spectrum scan reveals that CFO, TF, TF/CFO, and TF/CFO‐v samples predominantly comprise the elements of Fe, Cu, Ti, and O. The high‐resolution XPS spectrum of the Fe 2p orbit in Figure S7a exhibits peaks at 724.3, 718.7, and 710.8 eV, corresponding to the Fe 2p 3/2 orbit, Fe 2p 1/2 orbit, and its satellite peak, respectively. A shift towards higher binding energy in TF, TF/CFO, and TF/CFO‐v indicates an electron loss tendency in Fe post CFO binding. Furthermore, the low‐resolution XPS spectrum of the Cu 2p orbit in Figure 2e, displaying peaks at 953.55, 940.9, and 933.6 eV, aligns with the Cu 2p 3/2 orbit, Cu 2p 1/2 orbit, and its satellite peak. In accordance with Figure 2d, the binding energy of Cu moved to a higher value by approximately 0.3 eV upon combination with CFO, indicating an electron flow from TF to the CFO component in the composite sample. Furthermore, comparing the O1s spectra of TF and CFO in Figure S7b,c revealed that the binding energy of oxygen atoms decreased relative to CFO but increased relative to TF in the TF/CFO sample. This suggests an electron flow from TF to the CFO component in the composite sample, consistent with previous findings.

In order to decipher the impact of oxygen vacancies on the electronic properties of CuFeO_2_, density functional theory (DFT) calculations were performed. These calculations employed models of CuFeO_2_ slabs, both with and without oxygen vacancies, to simulate the material's electronic structure. The model presented in this study was developed based on the references and employed the (001) crystal plane of CuFeO_2_ [28, 29]. The introduction of oxygen vacancies notably manifested in the emergence of a new defect level proximal to the valence band maximum, as distinctly illustrated in Figure 2f,g. This defect level is indicative of a perturbation in the electronic structure due to the presence of vacancies. A detailed analysis of the partial charge density, presented in Figure 2h, reveals a significant electron accumulation around the valence band maximum in the oxygen vacancy‐incorporated CuFeO_2_ slab. This accumulation is markedly absent in the slab devoid of oxygen vacancies. The prevalence of these localized electrons near the valence band maximum is a crucial factor, as it facilitates the transfer of electrons into the anti‐bonding orbitals of CO_2_ molecules, thereby enhancing the activation process of CO_2_. This electronic redistribution, instigated by oxygen vacancies, underscores their pivotal role in altering the electronic landscape of CuFeO_2_, potentially leading to augmented catalytic activity.

**FIGURE 3:**
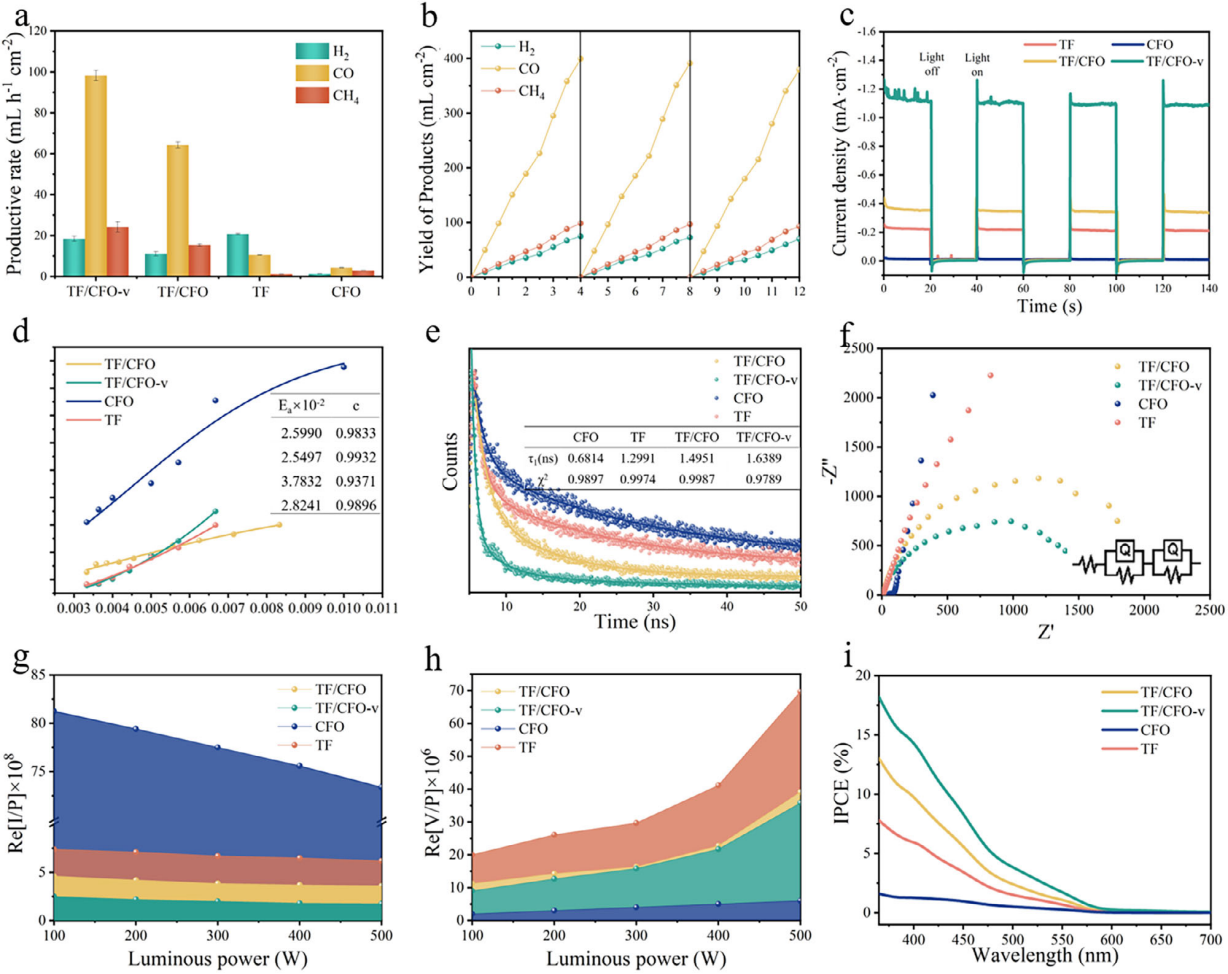
CO_2_ reduction (a) rate and (b) stability test of CFO, TF, TF/CFO and TF/CFO‐v. (c) Current‐time (I‐t) curves under chopped light illumination with a period of 20 s, (d) exciton binding energy, (e) transient photoluminescence spectrums, (f) EIS, (g) IMPS and (h) IMVS fit maps, (i)IPCE of the CFO, TF, TF/CFO and TF/CFO‐v.

Figure 3a,b display the CO_2_ reduction reaction (CO_2_RR) rates of the test samples under illumination and at an applied bias of −0.7 V. Notably, the TF/CFO‐v sample exhibited exceptional performance, generating H_2_, CO, and CH_4_ at rates of 18.41, 98.27, and 24.21 mL h^−1^, respectively. Here, CO emerged as the primary product, with production rates that are approximately 22.8, 9.4, and 1.5 times those of the CFO (4.31 mL h^−1^), TF (10.51 mL h^−1^), and TF/CFO (64.25 mL h^−1^) samples, respectively. Furthermore, after three cycles of 4‐h duration tests, the TF/CFO‐v sample consistently retained over 90% of its initial performance, demonstrating significant stability. Figure 3c presents the results from chopped light voltammetry tests, where the photocurrent densities for TF, CFO, TF/CFO, and TF/CFO‐v were −0.22, −0.02, −0.36, and −1.19 mA cm^−2^, respectively, indicating enhanced photoelectrocatalytic activity in the TF/CFO‐v sample. To elucidate the source of this improved performance, the light‐harvesting capabilities of the samples were first assessed (Figure S4). Compared with TF, the light absorption edges of TF/CFO and TF/CF‐V samples showed a red shift, indicating that CFO recombination expanded the light absorption range. Notably, the CFO sample absorbs light from 200 to 800 nm, owing to its unique narrow bandgap characteristics, which cover an extensive absorption spectrum. Subsequent tests evaluated the carrier migration and separation capabilities of the samples (Figure 3d–h). Through the fitting slope of in situ variable‐temperature fluorescence spectra, the exciton binding energy for each sample could be determined (Figure S8). It was observed that the exciton binding energy of the TF/CFO sample was reduced compared to TF and CFO, indicating that the heterostructure facilitates carrier transport, with the smallest exciton binding energy observed in TF/CFO‐v (2.55 × 10^−2^), suggesting enhanced carrier excitation due to the introduction of oxygen vacancies. Both transient and steady‐state fluorescence spectroscopy revealed corresponding results, with steady‐state fluorescence spectra shown in Figure S4 indicating a sequential decrease in fluorescence intensity from CFO to TF, TF/CFO, and TF/CFO‐v, implying a corresponding decrease in photogenerated electron‐hole pair recombination efficiency, indicative of enhanced carrier separation due to the heterostructure construction. The transient fluorescence spectra in Figure 3e, calculated using exponential fitting, showed fluorescence lifetimes for CFO, TF, TF/CFO, and TF/CFO‐v of 0.68, 1.50, 1.30, and 1.64 ns, respectively. Compared to CFO, the fluorescence lifetime of the TF/CFO‐v sample increased by about 2.3 times, suggesting that the formation of the TF/CFO heterostructure, combined with the introduction of oxygen vacancies, accelerates the mass transfer of carriers. EIS test results also corroborated this, with the impedance of TF/CFO being lower than that of CFO and TF, and reaching the lowest point upon the introduction of oxygen vacancies in the TF/CFO‐v. Figure 3g,h, from IMPS and IMVS fitting plots at different modulation frequencies (as shown in Figure S9), allowed for the calculation of carrier lifetimes and separation efficiencies. Compared to other samples, the TF/CFO‐V sample had a higher carrier lifetime and corresponding carrier separation efficiency, indicating that the introduction of oxygen vacancies enhances carrier transport properties. These tests collectively demonstrate that the construction of the heterostructure initially enhances the sample's light‐harvesting capabilities, and more importantly, improves carrier transport and separation abilities. The introduction of oxygen vacancies also plays a role in promoting carrier transport, thereby improving the electrical polarization during photoelectrocatalytic reactions, and enhancing the sample's incident photon‐to‐current efficiency (IPCE). As can be observed in Figure 3i, the photoelectric conversion efficiency of the TF/CFO‐V sample was the highest, approximately 17.63%, which is about 23 times that of CFO (0.81%), significantly enhancing the photoelectrocatalytic CO_2_ reduction performance.

**FIGURE 4:**
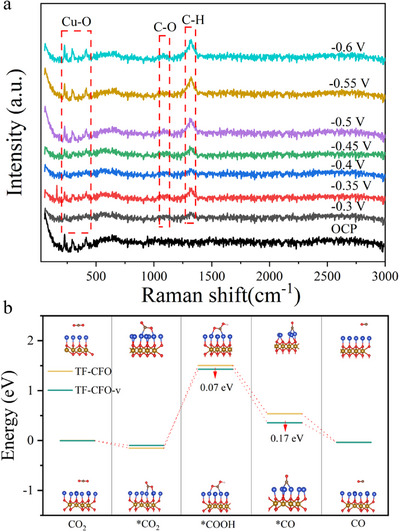
(a) In situ Raman spectroscopy of TF/CFO‐v, (b) step diagram of reaction path of CO_2_ conversion to CO.

Figure 4a shows the results of local Raman test of the sample in the CO_2_ reduction reaction. It can be seen that with the increase of voltage, the peak strength of C─O bond, C─H bond, and Cu─O bond gradually become stronger, indicating that the reaction is intensified with the increase of voltage. The C─O bond located near 1060 cm^−1^ represents the presence of carboxyl group, indicating that *COOH is the reaction intermediate, which provides a basis for determining the reaction path. Based on this, we have carried out the first principle simulation of the reaction process of CO_2_ reduction to CO. As show in Figure 4b, derived from meticulous first principle calculations, elegantly delineates the reaction mechanism for the reduction of CO_2_ to CO in CuFeO_2_, particularly highlighting the influence of oxygen vacancies (specific data are shown in Table S1, the schematic diagrams of the models used in the simulation calculation are shown in Figures S10 and S11). In the presence of these vacancies, CuFeO_2_ exhibits a significantly reduced energy barrier for the adsorption of *COOH, which is lowered by 0.07 eV compared to its oxygen vacancy‐free counterpart. This step is crucial as it represents the rate‐determining stage in the formation of CO [30]. The introduction of oxygen vacancies, therefore, plays a pivotal role in diminishing reaction barriers, thereby catalyzing the reduction process. Furthermore, the energy required for the subsequent adsorption of *CO is also notably decreased in the oxygen vacancy‐rich CuFeO_2_, thereby enhancing the likelihood of *CO desorption and streamlining the overall CO production pathway.

### 3.3 Relationship Between MHD Effect and Photoelectricity Performance

**FIGURE 5:**
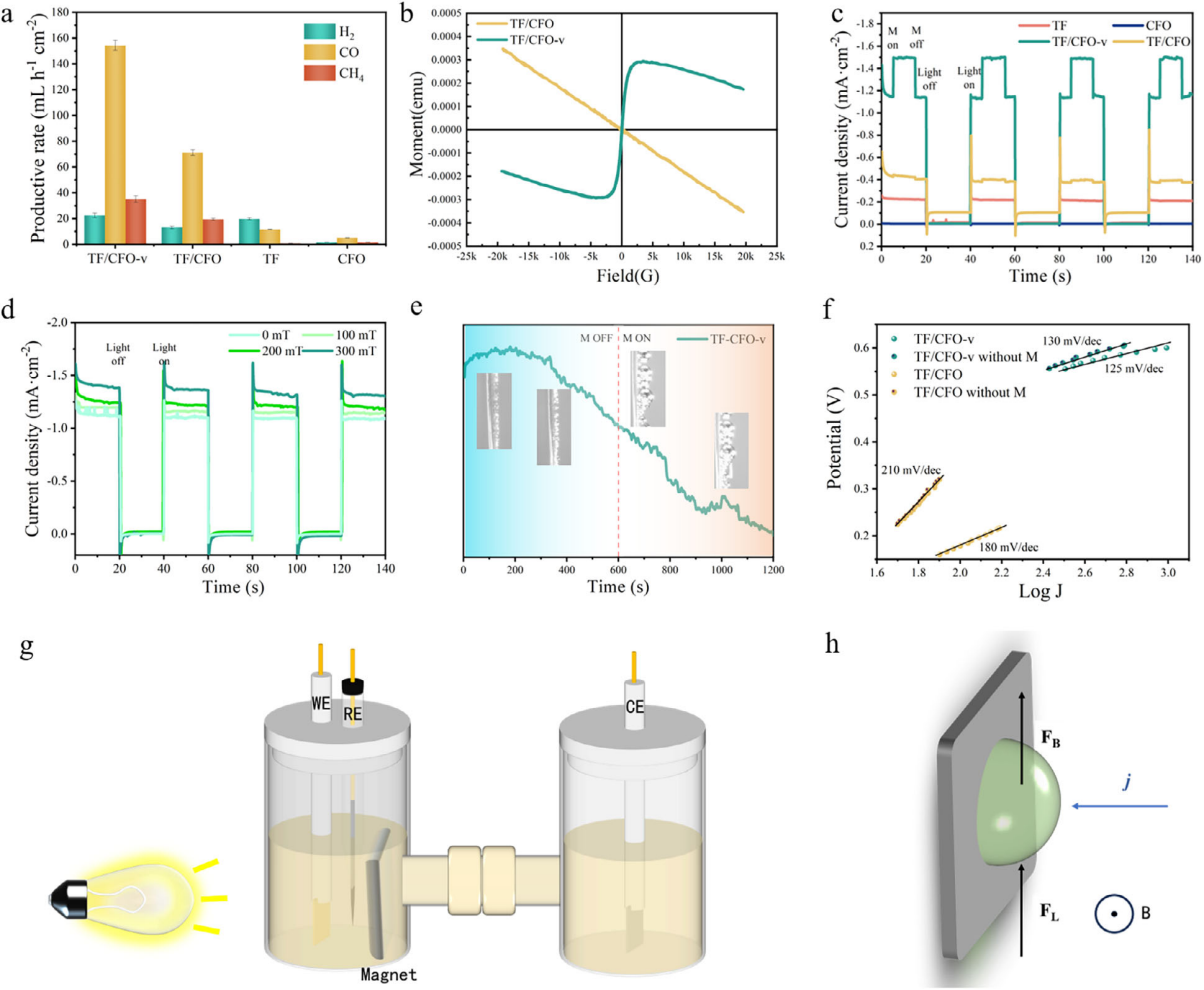
(a) Yield of CO_2_ reduction reaction under applied magnetic field, (b)VSM; Current–time (I‐t) curve under 20 s of cut‐off light irradiation under (c) 300 mT and (d) 100–300 mT magnetic field, (e) single high frequency impedance test, (f) Tafel, (g) diagram of CO_2_ reduction reaction under magnetic field, (h) schematic diagram of bubble force under magnetic fluid action.

Figure 5a shows the CO_2_ reduction reaction (CO_2_RR) generation rate of the tested sample under the application of a −0.7 V illumination and magnetic field. It is worth noting that TF/CFO‐v samples show excellent performance, generating H_2_, CO, and CH_4_ at 22.41, 154.27, and 35.21 mL h^−1^, respectively, which is about 1.2, 1.6, and 1.4 times higher than that without magnetic field. Here, CO becomes the main product, and Faraday efficiency reaches 38.97% (Figure S12). However, the differences in product yield are not statistically significant, indicating that the main function of the MHD effect is to enhance concentration polarization and catalytic efficiency, rather than significantly impacting the selectivity of CO_2_ reduction reaction products. In addition, it can be observed from the stability test shown in Figures S13–S18 that the sample still exhibits excellent stability under the conditions of light–electric–magnetic three fields. Figure 5b presents the vibrating sample magnetometer (VSM) results of the samples, demonstrating that those with oxygen vacancies exhibit notable weak ferromagnetic properties, in contrast to the non‐magnetic nature of samples without oxygen vacancies. This finding underscores the fundamental impact of oxygen vacancies on the magnetic properties of the samples. Figure S19 shows the fluorescence spectrum test of the sample under the influence of magnetic field. It can be seen from the figure that TF/CFO‐v containing oxygen vacancy decreases its fluorescence intensity under magnetic field due to its magnetic change. It is speculated that the dipole moment orientation of the sample with weak ferromagnetism tends to be consistent under the influence of magnetic field, thus inhibiting the recombination of carriers. Figure 5c depicts the chopped light‐magnetic voltammetry of the samples, revealing that under no magnetic field, the photocurrent density of the samples is similar to that in Figure 3c. However, upon magnetic field application, the current densities for TF/CFO‐v, TF/CFO, TF, and CFO samples are −1.49 (−1.13), −0.42 (−0.39), −0.22 (−0.22), −0.01 (−0.01) mA cm^−2^, respectively. This suggests that the introduction of a magnetic field benefits the photocurrent density of the magnetic TF/CFO‐v, presumably due to the alignment of magnetic moments enhancing charge carrier separation efficiency. Further, we explored the influence of magnetic field size on photocurrent density, as shown in Figure 5d, it can be seen that the light current density increases with the strengthening of the magnetic field, further confirming the correctness of the above inference. Figure 5e shows the single‐frequency impedance spectra of the samples, where, according to the curve and corresponding bubble overflow photos, the introduction of a magnetic field leads to larger bubbles due to the magnetohydrodynamic (MHD) effect, indicating an increase in the volume of expelled gas, and the enhancement of this performance is independent of the hydrophilicity or hydrophobicity of the sample itself (Figure S20). Figure 5f displays the Tafel plots of the samples, indicating no significant change in Tafel slope before and after magnetic field introduction, implying that the magnetic field has a minimal impact on charge carrier kinetics. Its main effect is due to the MHD effect. As shown in Figure 5h, under the action of MHD, the bubbles on the sample surface are accelerated from the upward force, thus reducing the thickness of the diffusion layer and accelerating the material transfer, thus improving the concentration polarization, and finally achieving the improvement of the performance of CO_2_RR. In addition, the performance of CO_2_RR is still in the top position in the comparison of related work in the industry (Figure S21).

The mechanism by which the MHD effect enhances the photocatalytic CO_2_ reduction reaction under the influence of a magnetic field (Scheme 1). In a manner akin to conventional photocatalytic processes, the semiconductor upon light absorption initiates a transition of electrons from the valence band. Driven by an external electric field, these electrons migrate to the surface, where they engage with CO_2_, yielding products such as CO and CH_4_. The key distinction here is that the MHD effect, induced by the magnetic field, leads to a reduction in the mass transfer distance of reactants on the surface and amplifies the desorption force of the gaseous products. Consequently, these dynamics facilitate an uptick in overall catalytic efficiency.

## 4 Conclusion

In conclusion, this study presents a comprehensive investigation into the kinetic improvement of CO_2_ reduction in O‐vacancy Fe_2_O_3_/CuFeO_2_ thin films catalyzed by the hydromagnetic effect. The research emphasizes the significant role of oxygen vacancies in enhancing the catalytic performance of CuFeO_2_, as evidenced by the reduced energy barrier for *COOH adsorption and improved *CO production. The study further explores the impact of magnetic fields on charge carrier dynamics, demonstrating an increase in photocurrent density and a marginal effect on carrier kinetics. These findings underscore the potential of integrating magnetic effects and oxygen vacancy engineering in optimizing photocatalytic CO_2_ reduction, offering promising avenues for efficient and sustainable carbon utilization technologies.

## Conflicts of Interest

The authors declare no conflicts interests.

## Supporting information




**Supporting Information file 1**: exp270100‐sup‐0001‐SuppMat.docx.
