# Supplementary material for: Synergistic Effects of MHD Dynamics and Oxygen Vacancies on Electrode Polarization in Photoelectrocatalysis CO2 Reduction Systems
Source: Exploration (Beijing). 2025 Dec 8;5(6):20240243. doi: 10.1002/EXP.20240243 (PMC12752557; doi:10.1002/EXP.20240243)
Supplement: Supplementary file 1 — Supporting Information file 1: exp270100‐sup‐0001‐SuppMat.docx. [file EXP2-5-20240243-s001.docx]

Supporting Information of :

**Synergistic Effects of MHD Dynamics and Oxygen Vacancies on Electrode Polarization in** **Photoelectrocatalysis CO_2_ Reduction Systems**

Lei Zhao^a^, Feng Xiao^a^, Xianghui Zeng^a^, Zhaohui Huang^b^, Wei Fang^a^, Xing Du^a^, Xuan He^a^, Weixin Li^a^, Daheng Wang^a^, Hui Chen^a, *^

^a.^ *The State Key Laboratory of Refractories and Metallurgy, Wuhan University of Science & Technology, Wuhan 430081, P.R. China.*

^b.^ *College of Materials Science and Engineering, Hunan University, P. R. China.*

**^*^** Corresponding author: Hui Chen, E-mail: [chenhui86@wust.edu.cn](mailto:chenhui86@wust.edu.cn)

Present address: 947 Heping Avenue, Qingshan District, Wuhan 430081, Hubei, P.R. China

**1.Experimental section**

**1.1 Preparation of Ti:Fe_2_O_3_(TF) film**

All regents were analytically pure and used without subsequent treatment. First, FTO-coated glass substrates were ultrasound in a mixture of acetone ethanol and deionized water (mass ratio 1:1:1) for 15 min. Then, 4.5μmol Ti_3_C_2_, 3mmol iron(III) chloride hexahydrate and equimolar urea were dissolved in 50 mL DI water to form a homogeneous precursor solution. Thereafter, substrates were placed in 100 mL Teflon-lined autoclave, with FTO side facing down. The precursor solution was then poured into the autoclave container. The reaction was carried out at 95 ºC for 5 h in the oven, forming a uniform yellowish Ti:FeOOH film. The Ti:FeOOH film was heat treated in a tube furnace at 550 °C for 2 h in air with a heating rate of 10 °C/min, and then the temperature was further raised to 750 °C at a heating rate of 20 °C/min for 15 min. Consequently, a red-orange Ti:Fe_2_O_3_ film was obtained, named TF.

**1.2 Preparation of CuFeO_2_(CFO) film**

The CuFeO_2_ precursor solution was formed by mixing 4 mmol CuSO_4_, 4 mmol Fe_2_(SO_4_)_3_ and 8 mmol citric acid, adding 20 mL ethanol and stirring for 10 h, and then adding 2 mL ethylene glycol and stirring again overnight. The suspension coating method was used to take 60ul of precursor solution and drop it on FTO at a rotational speed of 3500 rpm/min for a suspension coating time of 30 s. After that, it was dried at 100 °C for 1 h, and this step was repeated for 6-12 times. Finally, it was roasted at 550 °C for 2 h to obtain CuFeO_2_ film, named CFO.

**1.3 Preparation of Ti:Fe_2_O_3_/CuFeO_2_(TF/CFO) film**

The CuFeO_2_ precursor solution prepared in 1.2 was suspended on Ti:FeOOH prepared in 1.1. After drying at 100 °C for 1h, the suspension coating step was repeated 1-3 times, and then the Ti:Fe_2_O_3_/CuFeO_2_ film was obtained by roasting it at 550 °C for 2h, named TF/CFO

**1.4 Preparation of Ti:Fe_2_O_3_/CuFeO_2_-v(TF/CFO-v) film**

The Ti:Fe_2_O_3_/CuFeO_2_ film prepared in 1.3 was annealed for 2h at 300 ° C under Ar/H_2_ atmosphere and cooled to room temperature naturally to obtain the sample Ti:Fe_2_O_3_/CuFeO_2_-v, named TF/CFO-v.

**2.Result**


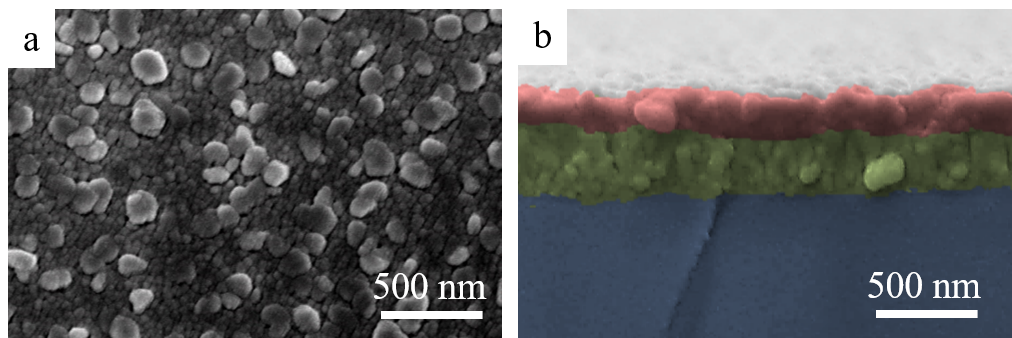


**Fig. S1**. (a)SEM top view and (b) SEM cross-section photo of CFO.


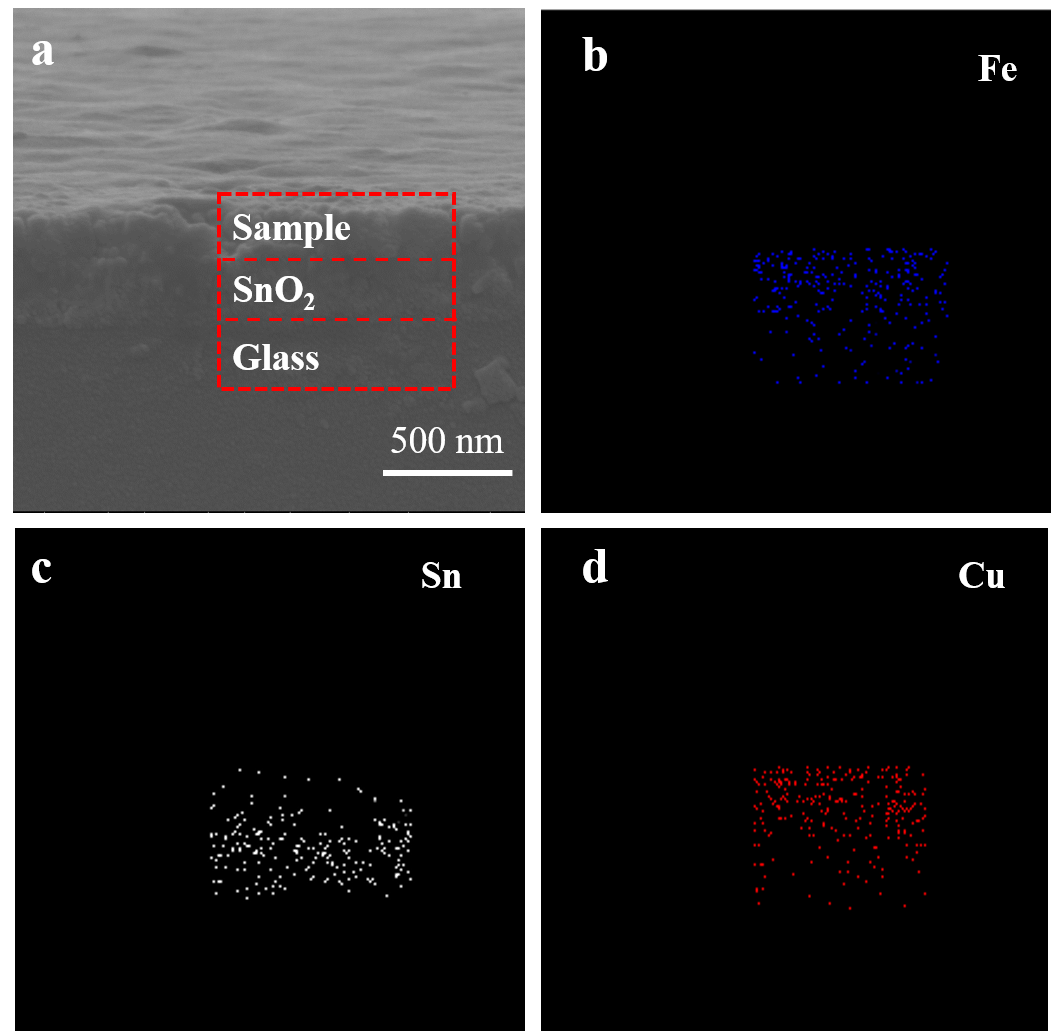


**Fig. S2**. (a)SEM photo and (b-d)energy spectrum of TF/CFO-v.


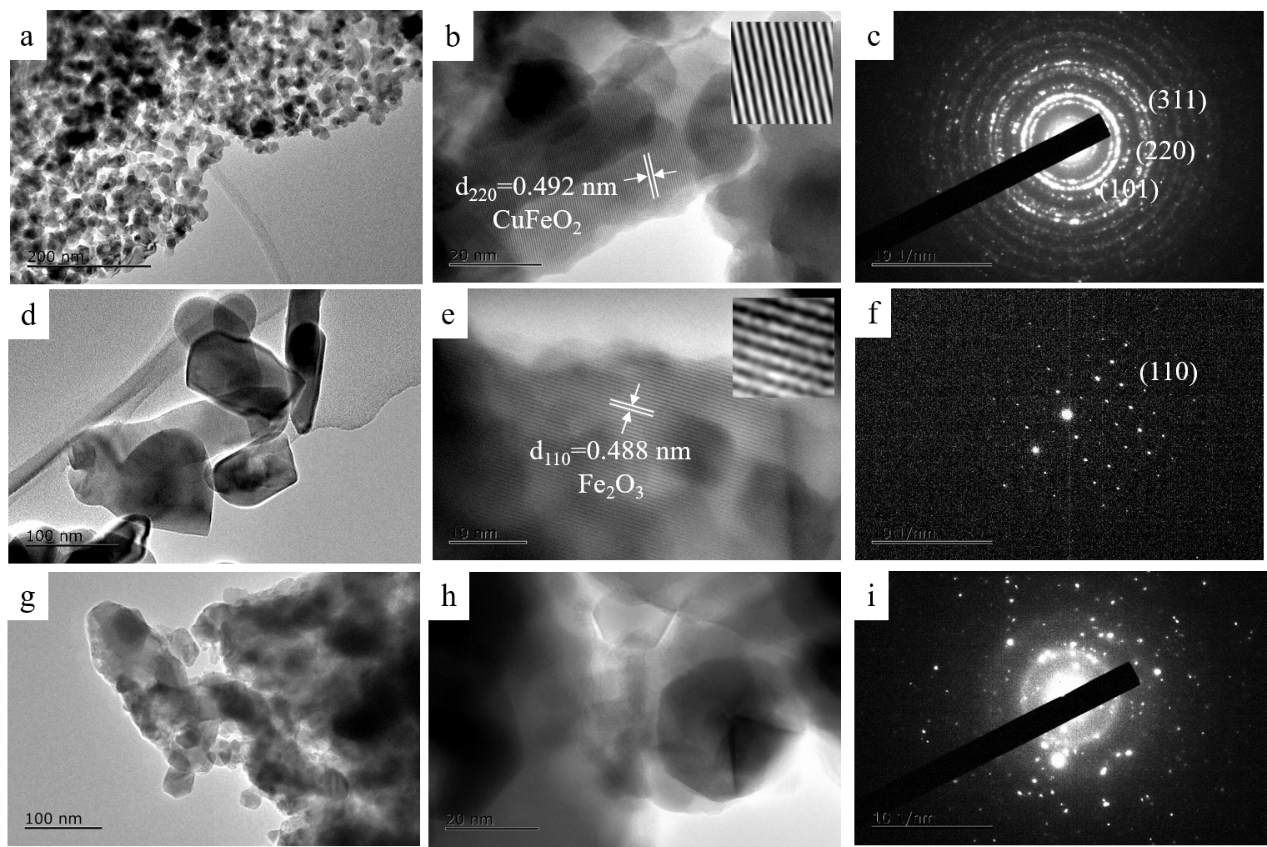


**Fig. S3**. (a,d,g) TEM photo ,(b,e,h) HRTEM photo and (c,f,i) diffraction pattern of CFO, TF, TF/CFO;.


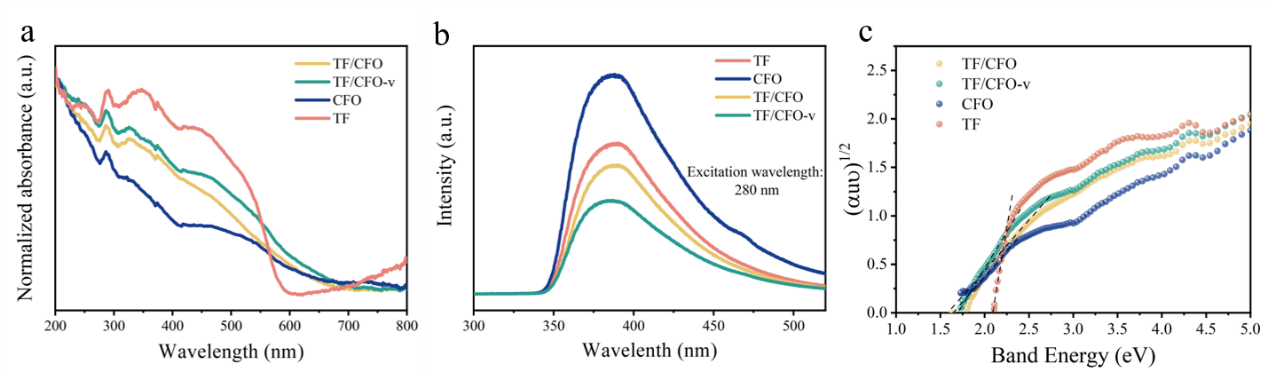


**Fig. S4**. (a) UV−Vis absorbance spectra, (b) the steady state photoluminescence spectra and (c) Tauc plot of the CFO, TF, TF/CFO and TF/CFO-v.





**Fig. S5**. The Mott-Schottky(MS) curve of samples


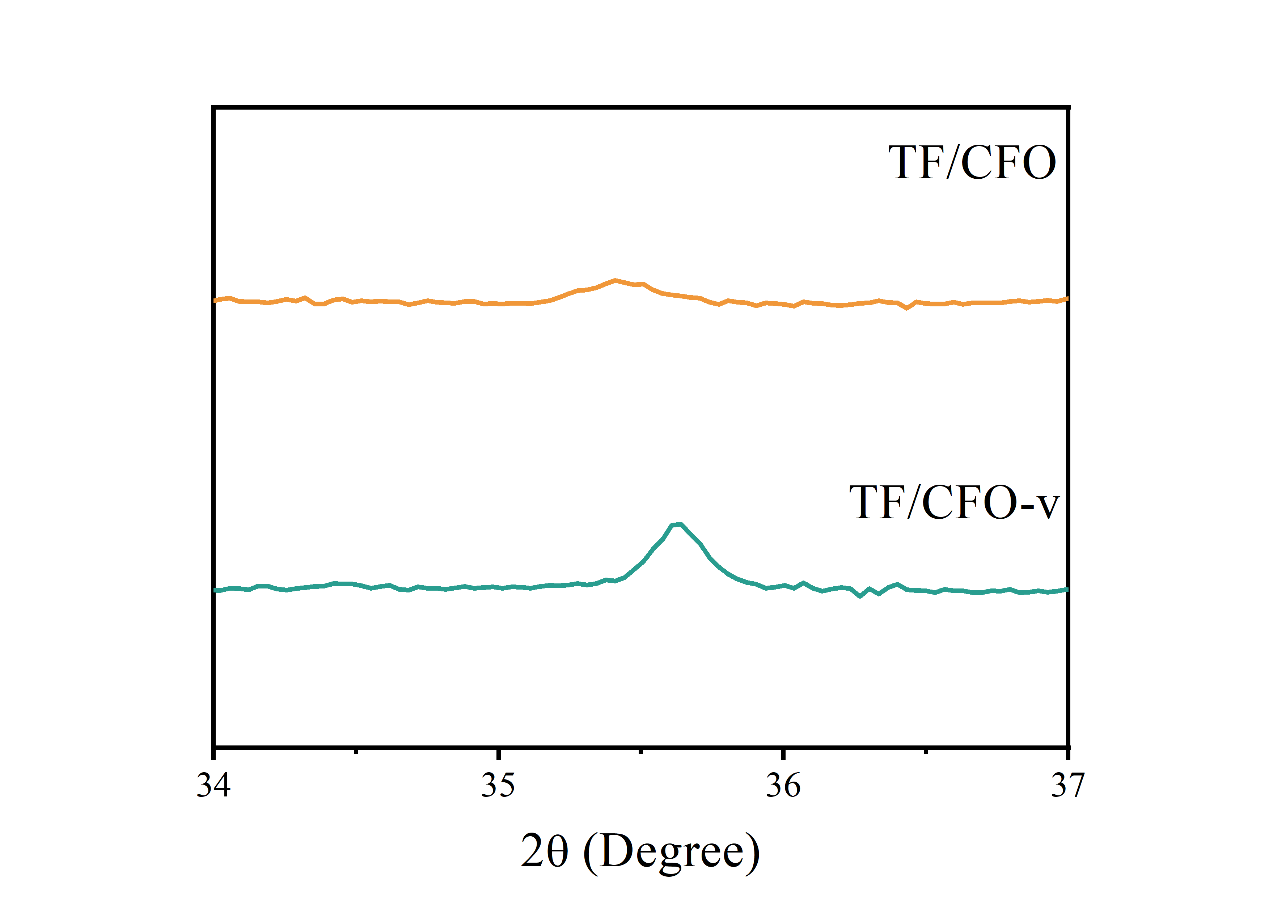


**Fig. S6**. XRD spectrum of TF/CFO and TF/CFO-v.


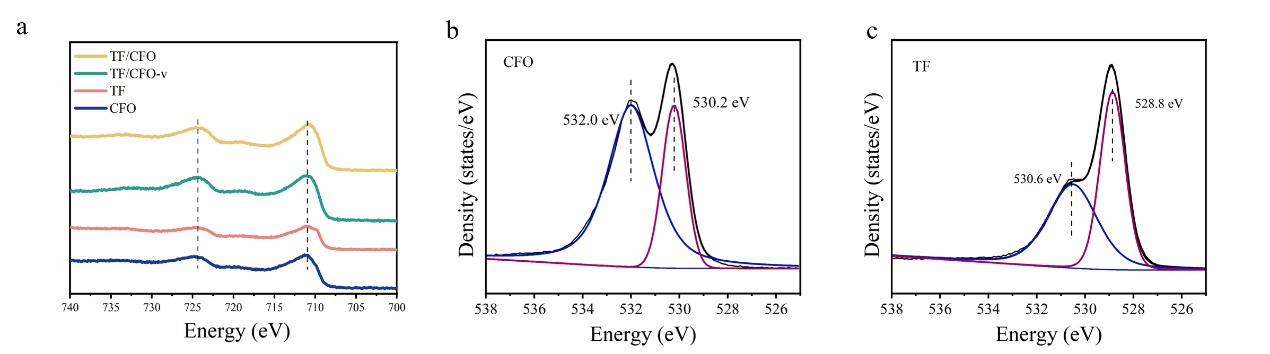


**Fig. S7.** XPS (a) Fe 2p and (b, c)O 1s spectrum of samples


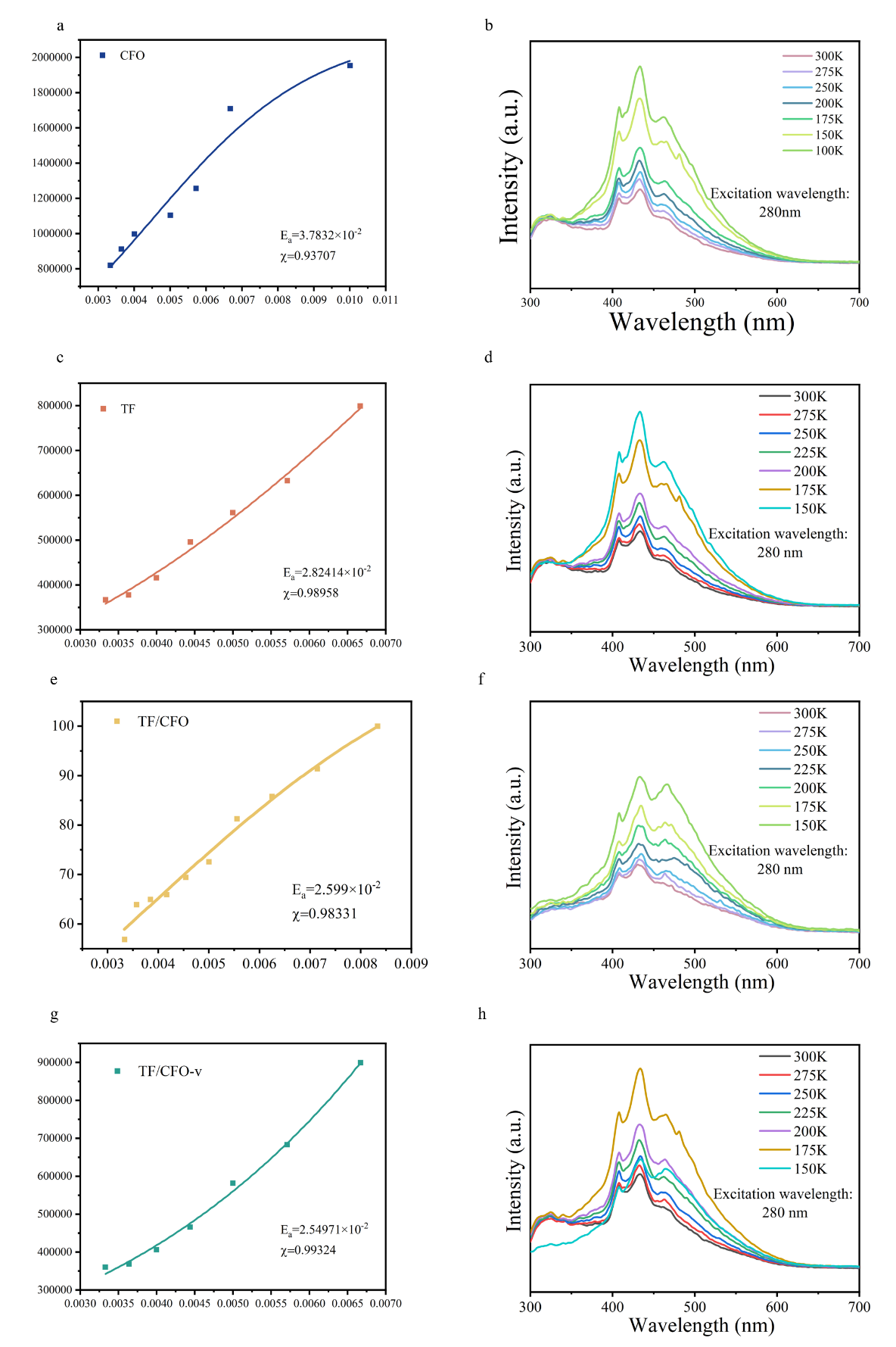


**Fig.S8.** Variable temperature fluorescence spectrum.


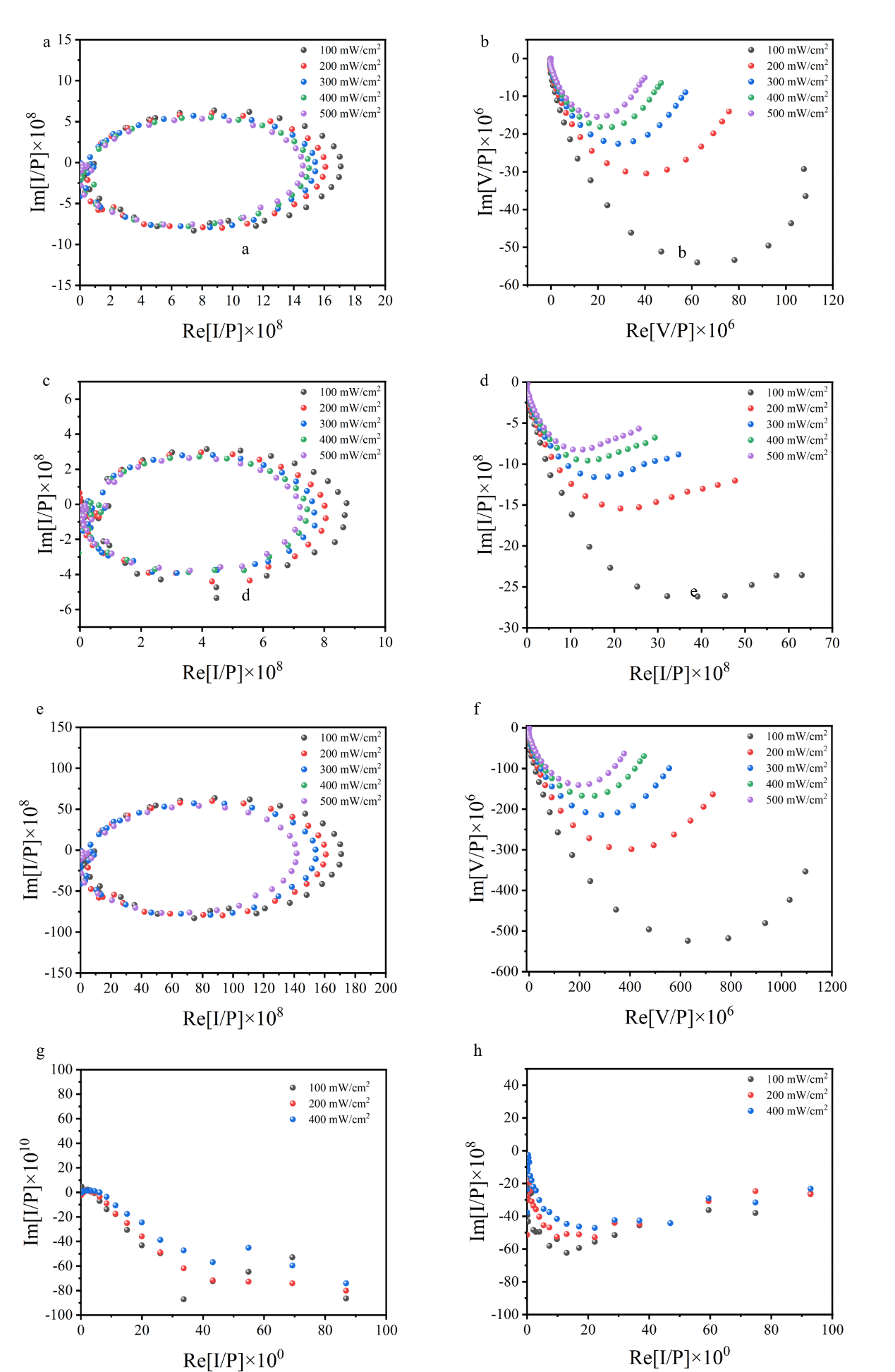


**Fig. S9**. (a,c,e,g) IMPS and (b,d,f,h) IMVS of TF/CFO-v, TF/CFO, TF and CFO


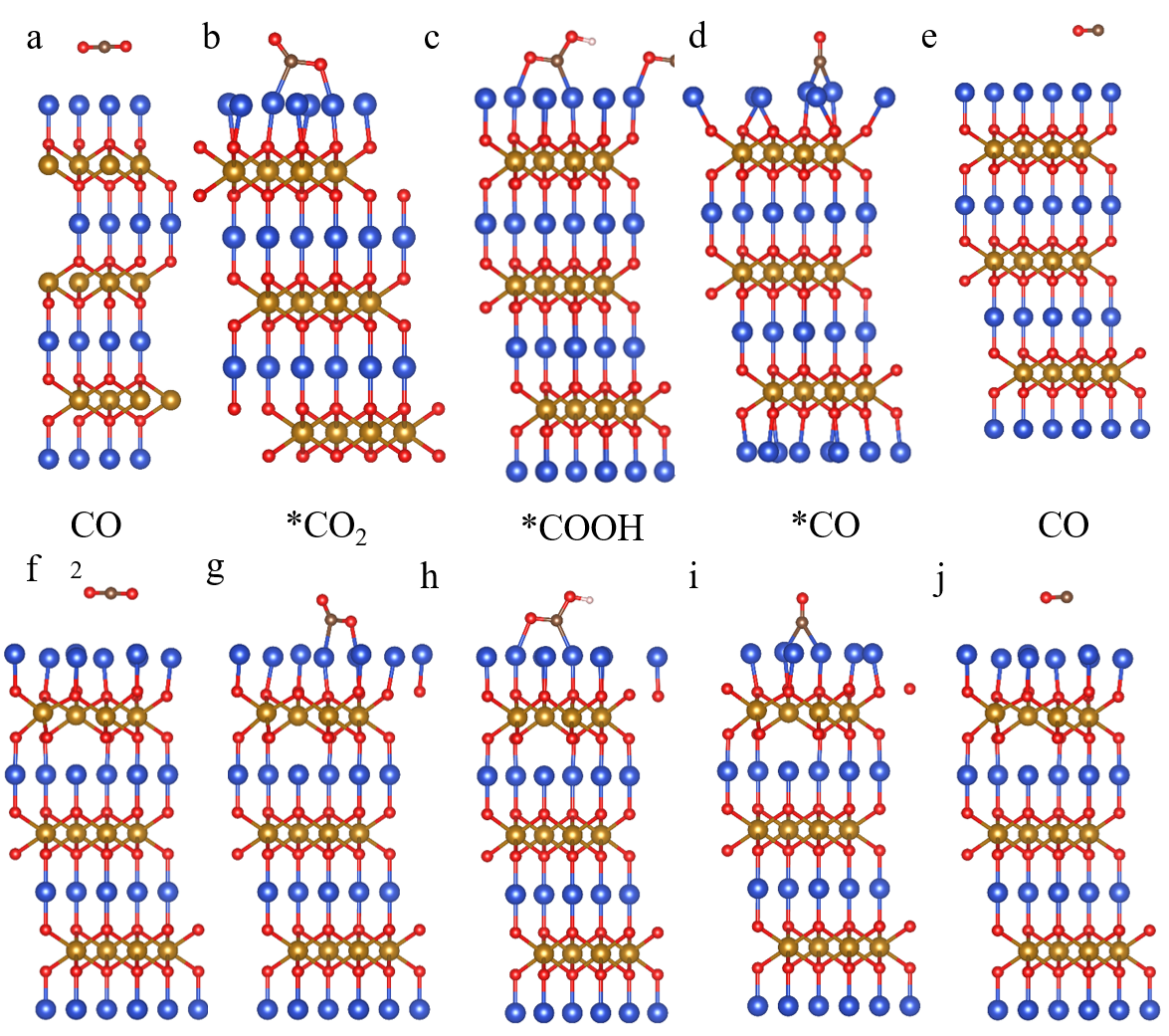


**Fig. S10**. Structural simulation of CO_2_ reduction to CO in (a-e)bulk phase CuFeO_2_ and (f-j)CuFeO_2_-v.


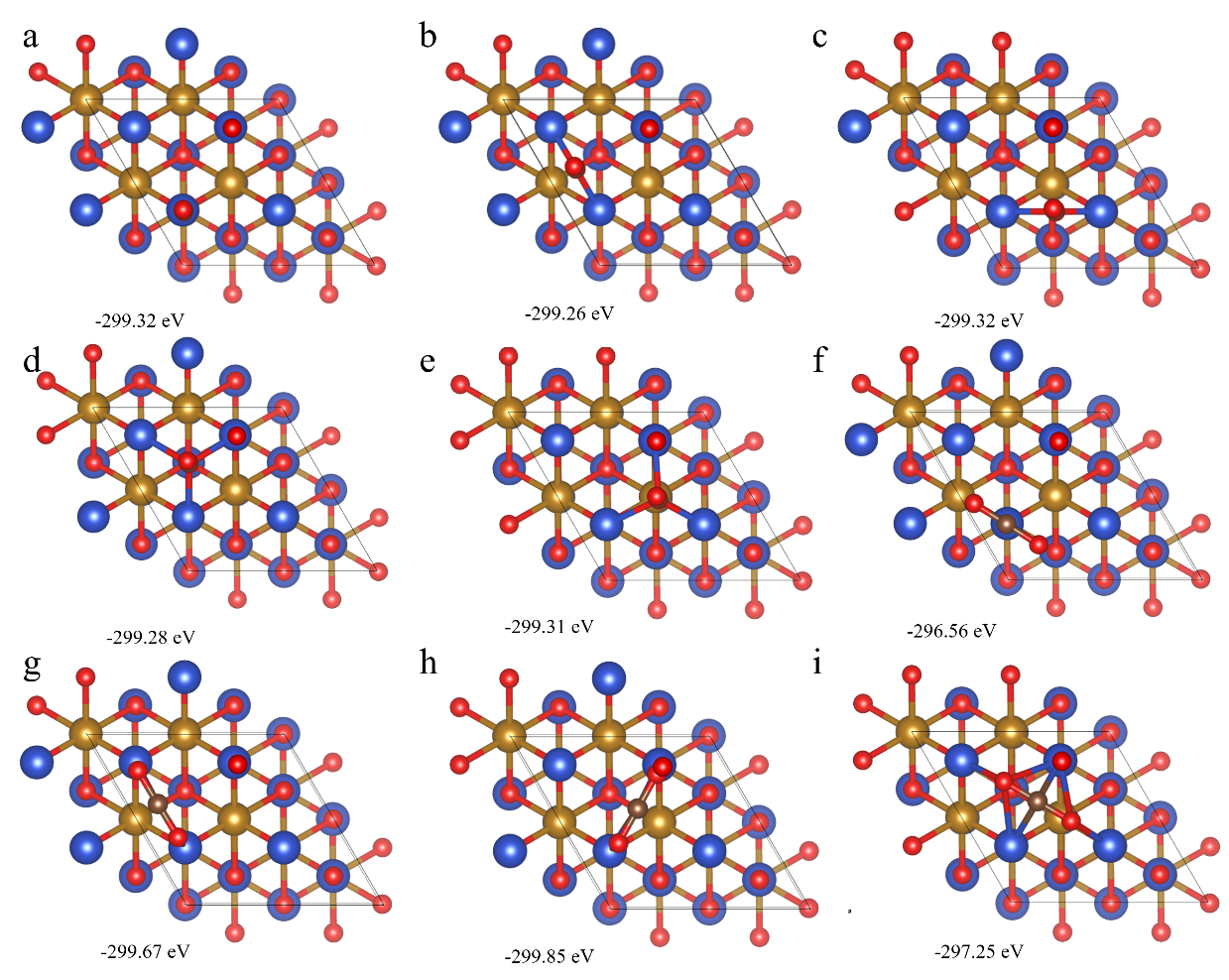


**Fig. S11**. Adsorption models of carbon dioxide on different sites of CuFeO_2_ surface


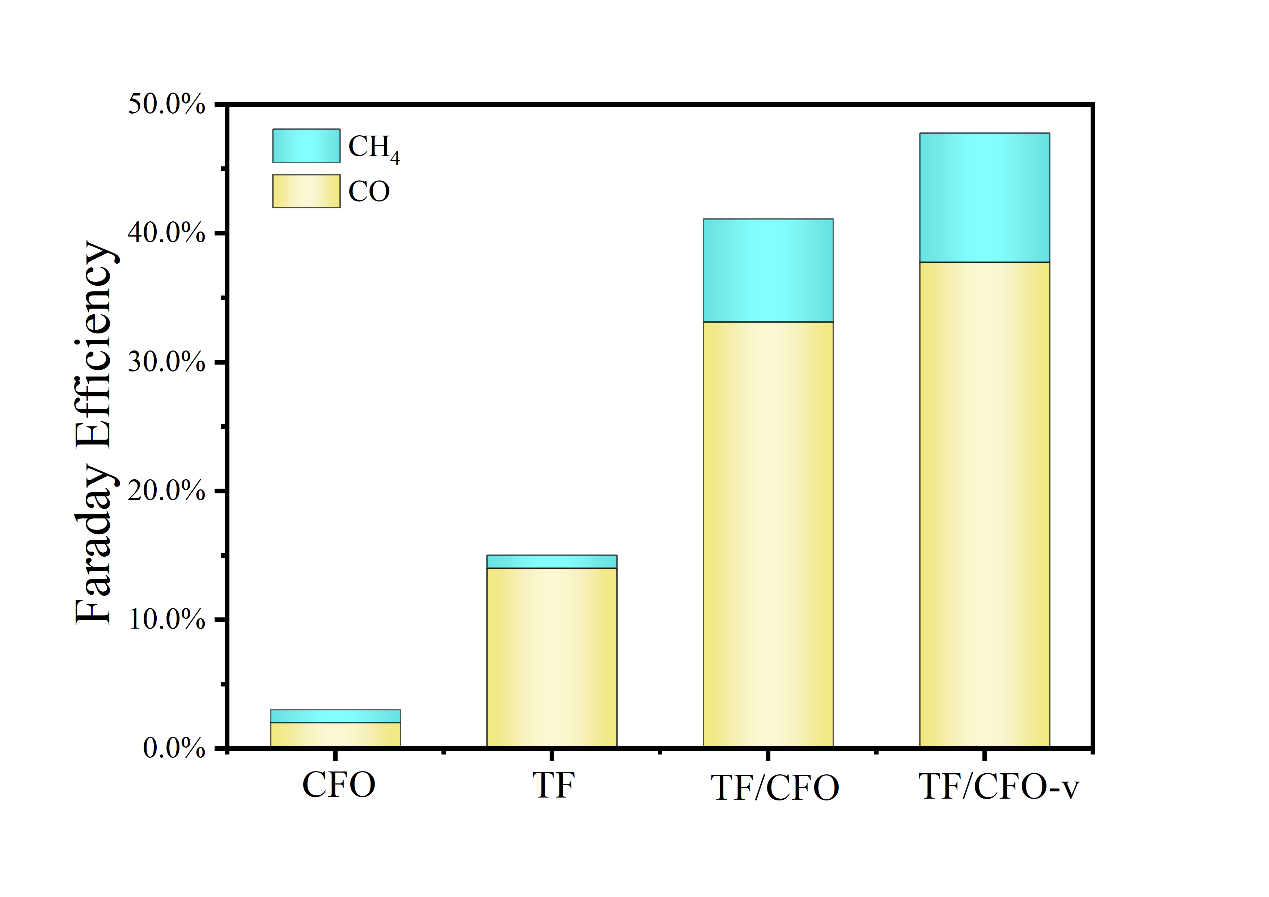


**Fig.S12**. The FE test of samples





**Fig.S13**. Stability testing without magnetic conditions.


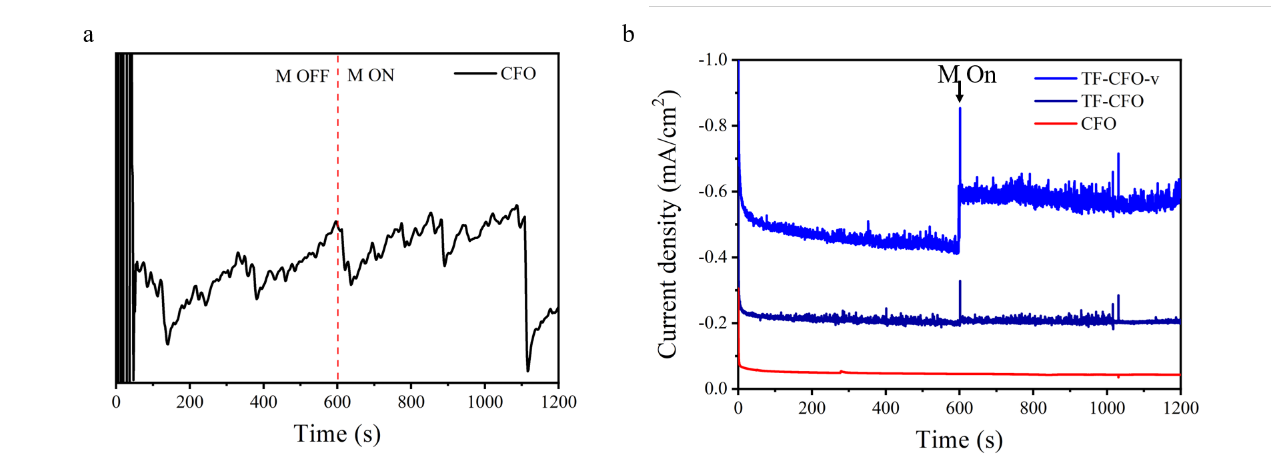


**Fig.S14**. (a) Single high frequency impedance test of CFO, (b) Stability testing under magnetic conditions


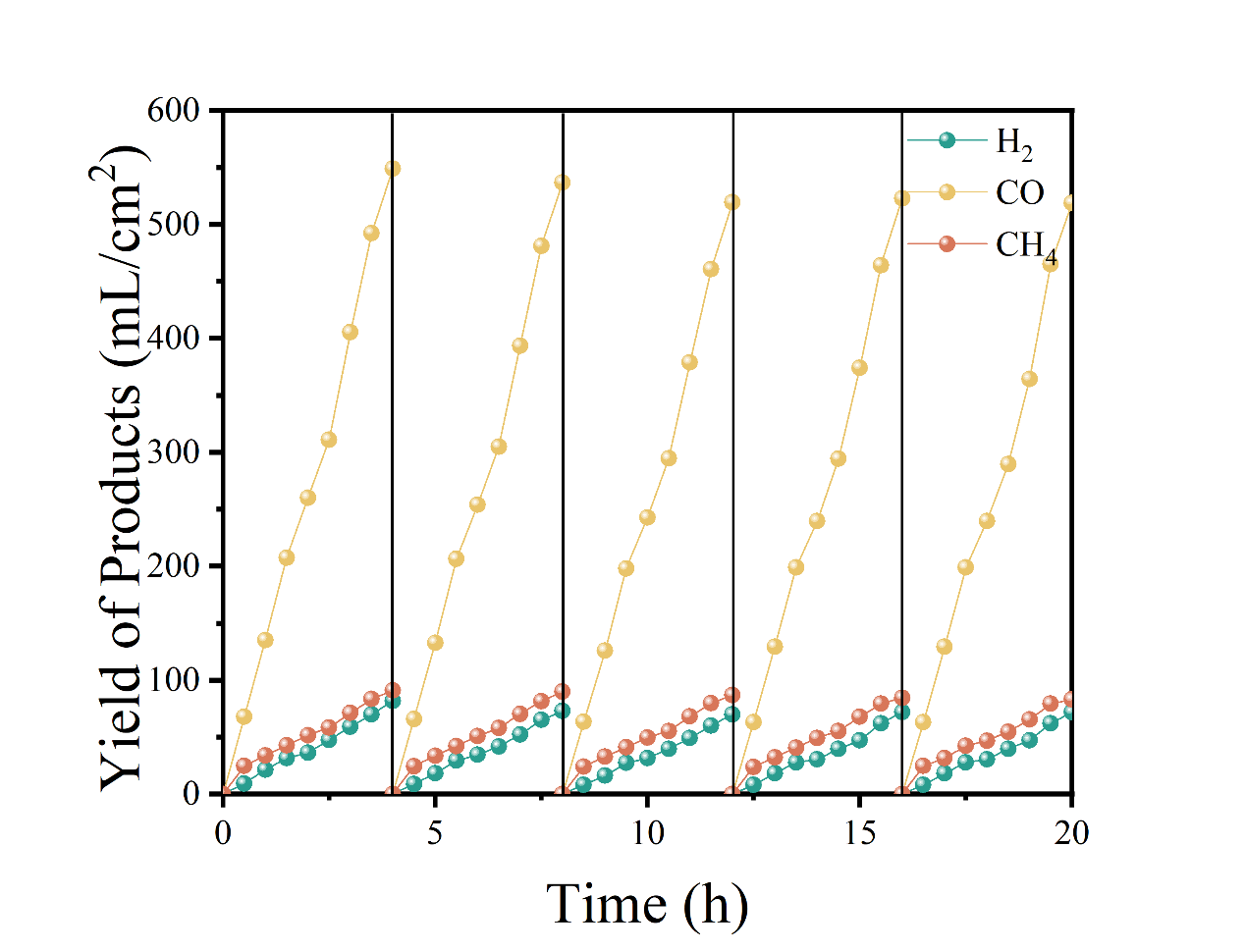


**Fig.S15**. Stability testing under magnetic conditions of TF-CFO-v





**Fig. S16**. XRD results of TF-CFO-v before and after CO2RR


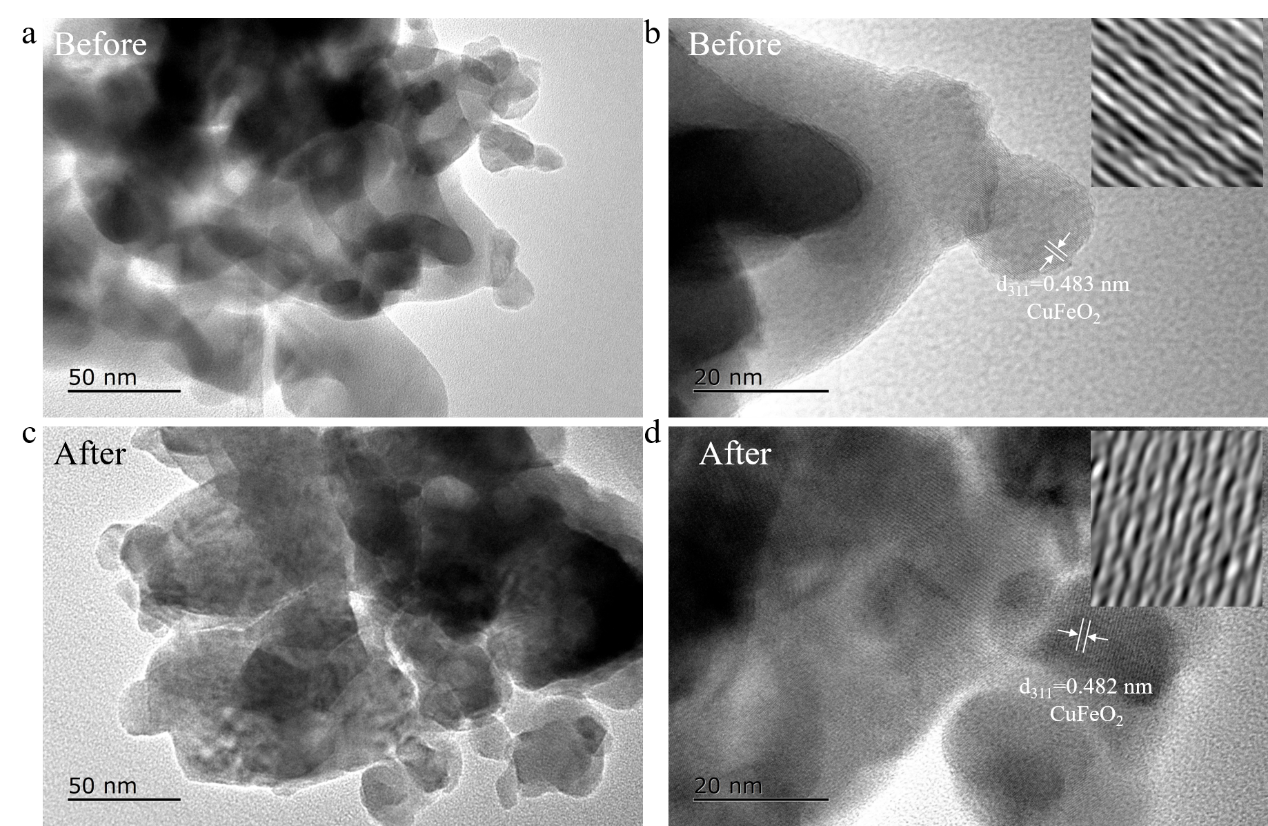


**Fig. S17**. TEM photos of TF-CFO-v (a, b) before and (c, d) after CO_2_RR


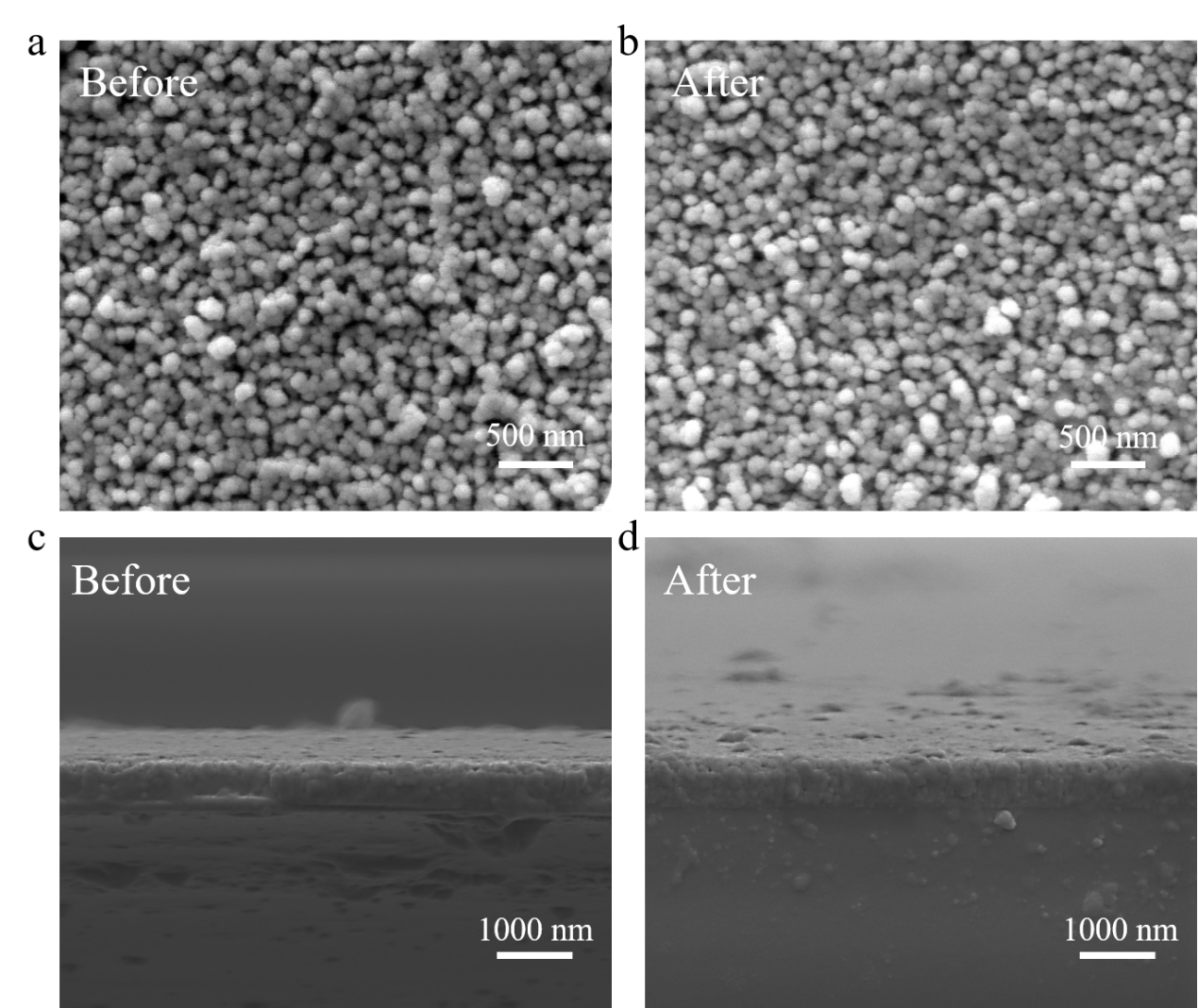


**Fig. S18**. SEM photos of TF-CFO-v (a, c) before and(b, d) after CO_2_RR


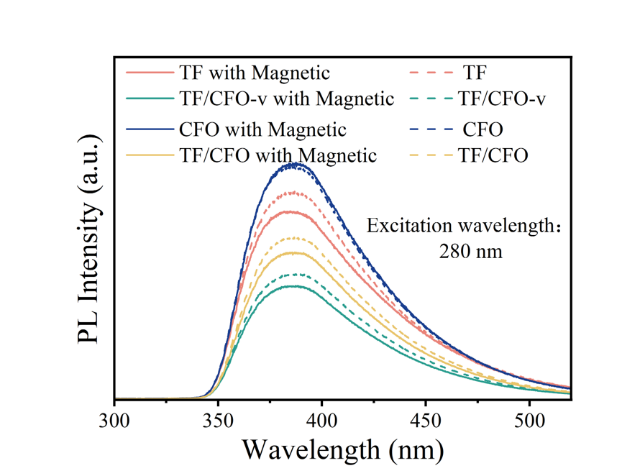


**Fig.S19**. Fluorescence spectra under the influence of 300 mT magnetic field


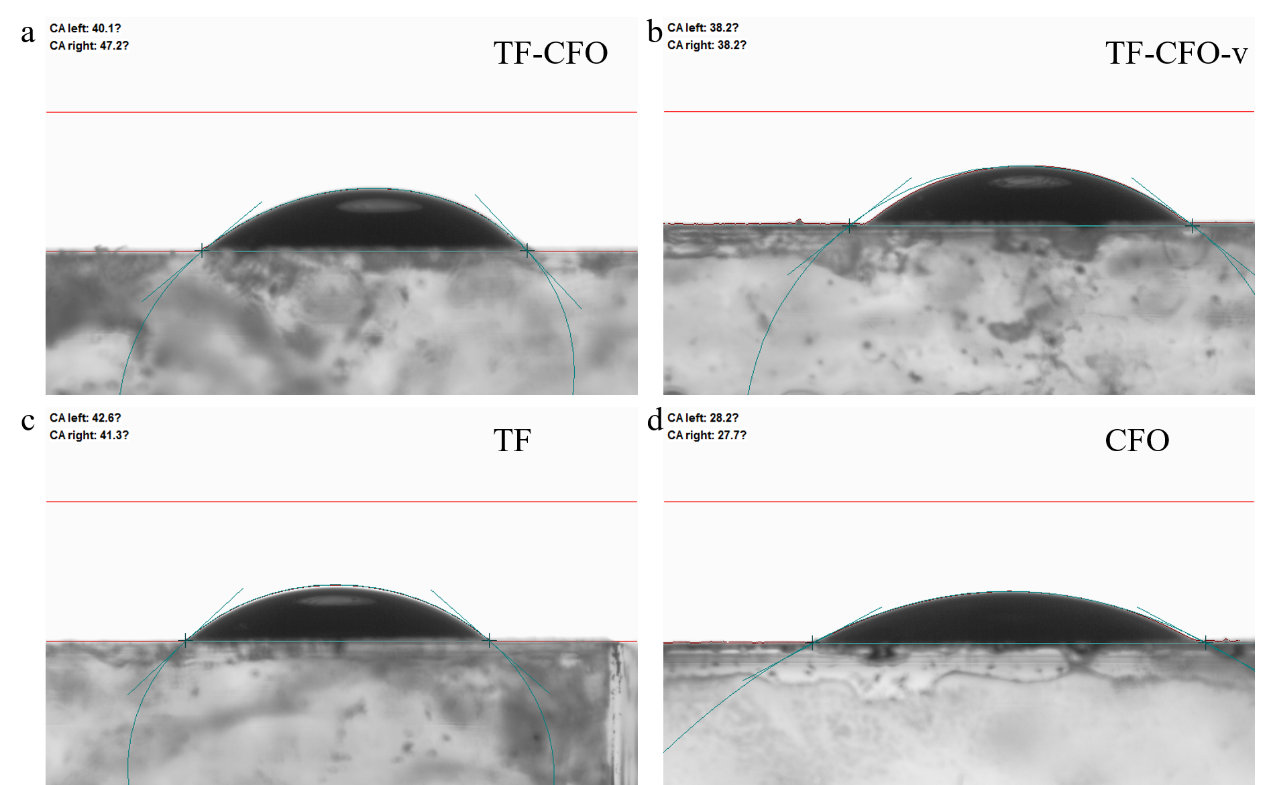


**Fig.S20**. Contact angle test results of (a) TF-CFO, (b) TF-CFO-v, (c) TF and (d) CFO


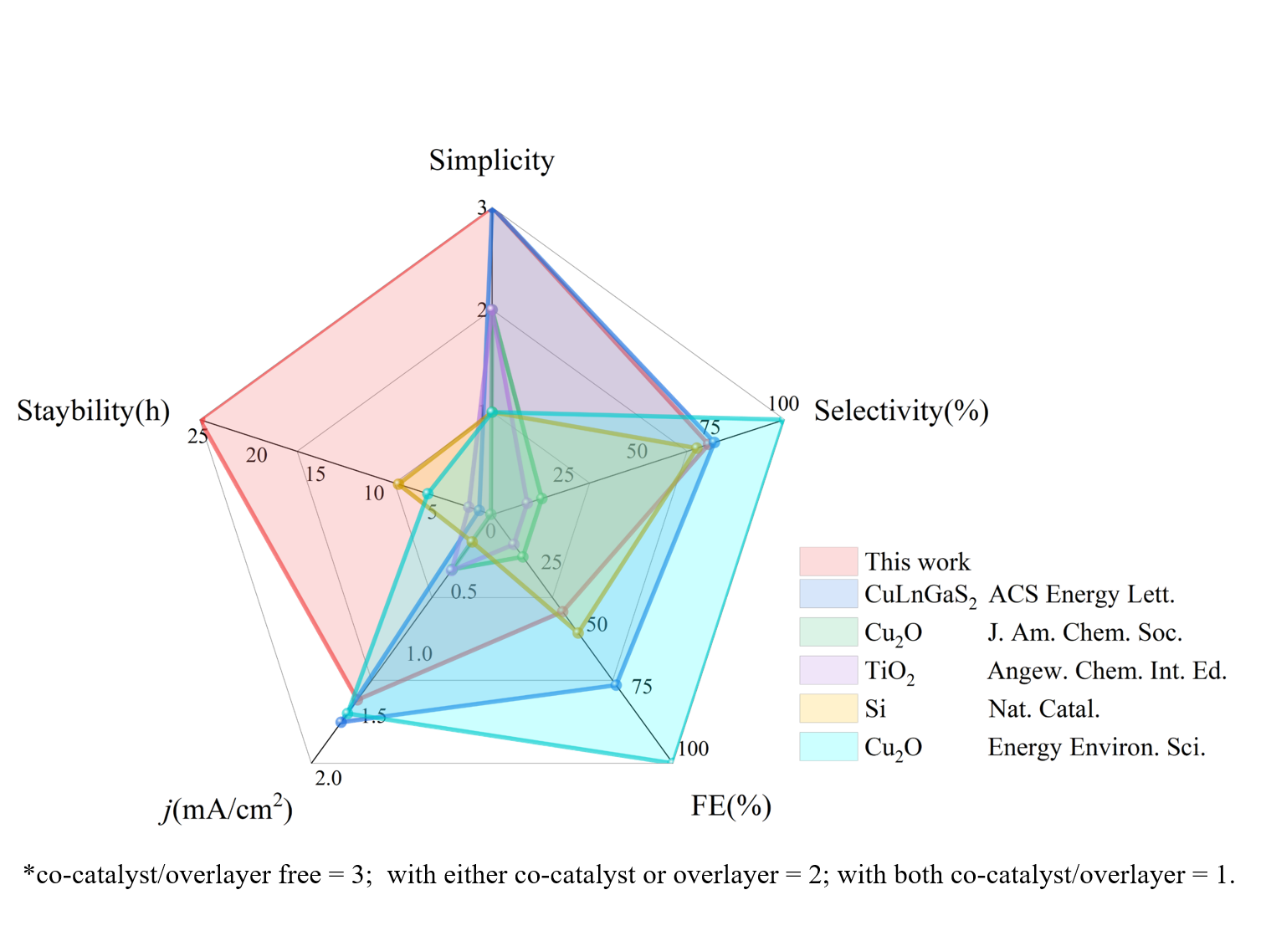


**Fig. S21.** Structural simulation of CO_2_^[1-5]^

Table S1 Each intermediate step of the reaction corresponds to the energy

|  | CO_2_ | *CO_2_ | *COOH | *CO | CO |
| --- | --- | --- | --- | --- | --- |
| TF-CFO | 0.0000 | -0.1539 | 1.5037 | 0.5359 | -0.0358 |
| TF-CFO-v | 0.0000 | -0.0963 | 1.4336 | 0.3597 | -0.0358 |

**3.References**

[1] Liu Y, Xia M, Ren D, et al. Photoelectrochemical CO_2_ Reduction at a Direct CuInGaS_2_/Electrolyte Junction[J]. ACS Energy Letters, 2023, 8(4): 1645-1651.

[2] Deng X, Li R, Wu S, et al. Metal–organic framework coating enhances the performance of Cu_2_O in photoelectrochemical CO_2_ reduction[J]. Journal of the American Chemical Society, 2019, 141(27): 10924-10929.

[3] Rosser T E, Windle C D, Reisner E. Electrocatalytic and Solar‐Driven CO_2_ Reduction to CO with a Molecular Manganese Catalyst Immobilized on Mesoporous TiO_2_[J]. Angewandte Chemie International Edition, 2016, 55(26): 7388-7392.

[4] Leung J J, Warnan J, Ly K H, et al. Solar-driven reduction of aqueous CO_2_ with a cobalt bis (terpyridine)-based photocathode[J]. Nature Catalysis, 2019, 2(4): 354-365.

[5] Chang X, Wang T, Gong J. CO_2_ photo-reduction: insights into CO_2_ activation and reaction on surfaces of photocatalysts[J]. Energy & Environmental Science, 2016, 9(7): 2177-2196.
